# Arbuscular mycorrhizal fungi impact the production of alkannin/shikonin and their derivatives in *Alkanna tinctoria* Tausch. grown in semi-hydroponic and pot cultivation systems

**DOI:** 10.3389/fmicb.2023.1216029

**Published:** 2023-08-10

**Authors:** Yanyan Zhao, Annalisa Cartabia, Mónica Garcés-Ruiz, Marie-France Herent, Joëlle Quetin-Leclercq, Sergio Ortiz, Stéphane Declerck, Ismahen Lalaymia

**Affiliations:** ^1^Mycology, Earth and Life Institute, Université catholique de Louvain – UCLouvain, Louvain-la-Neuve, Belgium; ^2^Pharmacognosy Research Group, Louvain Drug Research Institute (LDRI), Université catholique de Louvain – UCLouvain, Brussels, Belgium; ^3^UMR 7200, Laboratoire d’Innovation Thérapeutique, Université de Strasbourg, CNRS, Strasbourg Drug Discovery and Development Institute (IMS), Illkirch-Graffenstaden, France

**Keywords:** arbuscular mycorrhizal fungi, *Alkanna tinctoria*, semi-hydroponic cultivation system, alkannin/shikonin derivatives, native strains

## Abstract

**Introduction:**

*Alkanna tinctoria* Tausch. is a medicinal plant well-known to produce important therapeutic compounds, such as alkannin/shikonin and their derivatives (A/Sd). It associates with arbuscular mycorrhizal fungi (AMF), which are known, amongst others beneficial effects, to modulate the plant secondary metabolites (SMs) biosynthesis. However, to the best of our knowledge, no study on the effects of AMF strains on the growth and production of A/Sd in *A. tinctoria* has been reported in the literature.

**Methods:**

Here, three experiments were conducted. In Experiment 1, plants were associated with the GINCO strain *Rhizophagus irregularis* MUCL 41833 and, in Experiment 2, with two strains of GINCO (*R. irregularis* MUCL 41833 and *Rhizophagus aggregatus* MUCL 49408) and two native strains isolated from wild growing *A. tinctoria* (*R. irregularis* and *Septoglomus viscosum*) and were grown in a semi-hydroponic (S-H) cultivation system. Plants were harvested after 9 and 37 days in Experiment 1 and 9 days in Experiment 2. In Experiment 3, plants were associated with the two native AMF strains and with *R. irregularis* MUCL 41833 and were grown for 85 days in pots under greenhouse conditions. Quantification and identification of A/Sd were performed by HPLC-PDA and by HPLC-HRMS/MS, respectively. *LePGT1*, *LePGT2*, and *GHQH* genes involved in the A/Sd biosynthesis were analyzed through RT-qPCR.

**Results:**

In Experiment 1, no significant differences were noticed in the production of A/Sd. Conversely, in Experiments 2 and 3, plants associated with the native AMF *R. irregularis* had the highest content of total A/Sd expressed as shikonin equivalent. In Experiment 1, a significantly higher relative expression of both *LePGT1* and *LePGT2* was observed in plants inoculated with *R. irregularis* MUCL 41833 compared with control plants after 37 days in the S-H cultivation system. Similarly, a significantly higher relative expression of *LePGT2* in plants inoculated with *R. irregularis* MUCL 41833 was noticed after 9 versus 37 days in the S-H cultivation system. In Experiment 2, a significant lower relative expression of *LePGT2* was observed in native AMF *R. irregularis* inoculated plants compared to the control.

**Discussion:**

Overall, our study showed that the native *R. irregularis* strain increased A/Sd production in *A. tinctoria* regardless of the growing system used, further suggesting that the inoculation of native/best performing AMF is a promising method to improve the production of important SMs.

## 1. Introduction

The hydroxynaphthoquinones (HNQs) of natural origin, and in particular the isohexenylnaphthazarins (IHNs), such as the chiral pair alkannin and shikonin (A/S), are lipophilic red pigments characterized by a wide spectrum of wound healing, antibacterial, anti-inflammatory, anticancer, and antithrombotic activities (Papageorgiou et al., 1999; Tappeiner et al., 2014). Monomeric A/S derivatives, mainly esters of the side chain hydroxyl group, have been found in the root periderm of several plants of the Boraginaceae family, including *Alkanna tinctoria* Tausch. This important medicinal plant, commonly known as alkanet or dyers’ bugloss/alkanet, is found across southern Europe, northern Africa, and southwestern Asia, with a central distribution in the Mediterranean region (Valdés, 2011). Preparations made with *A. tinctoria* roots are commonly used in traditional medicine to treat wounds, burns, and ulcers, while extracted A/S are employed as active ingredients in different marketed pharmaceutical formulations (e.g., Histoplastin Red^®^ and HELIXDERM^®^) as strong wound healing medicines. Root extracts are also used as cosmetics, food additives or natural dyes for staining silk (Papageorgiou et al., 2008; Malik et al., 2016).

Today, nature remains the main source of commercial A/S and their derivatives (A/Sd), despite significant efforts to synthesize these compounds over the years (Papageorgiou et al., 2008). The only successful example of shikonin scaling up from cell cultures was with *Lithospermum erythrorhizon* Siebold & Zucc. (Boraginaceae) by Mitsui Petrochemical Industries Ltd. (now Mitsui Chemicals Inc., Tokyo, Japan) in 1984 (Yazaki, 2017). Few more attempts have been made with cell suspension cultures of *Arnebia* spp. (Boraginaceae) in stirred-tank (New Brunswick Scientific Company Inc., Edison, NJ, USA) and in air-lift bioreactors, but no further commercial successes have been reported (Gupta et al., 2014; Malik et al., 2016). For *A. tinctoria*, cell tissues and root cultures have been used, but the level of A/S production remained insufficient for commercial use (Urbanek et al., 1996; Gerardi et al., 1998). Yet, these important compounds remain mostly extracted from plants grown in the wild, with a risk of extinction as already documented for other Boraginaceae spp. (e.g., *L. erythrorhizon* in Japan, *Alkanna sieheana* Rech. Fil. and *Alkanna orientalis* L. Boiss. in Turkey). Indeed, *A. tinctoria* is considered as very rare in some European countries, such as Slovakia, Bulgaria, and Hungary (Yazaki, 2017; Yaman et al., 2019; Ahmad et al., 2021). Moreover, this plant is characterized by a very low seed germination rate and its cultivation under conventional agriculture practices is hampered by several constraints (e.g., time needed for A/S production, high costs of harvesting, and exposure to biotic/abiotic stresses) (Malik et al., 2016). For these reasons, *in vitro* and *ex vitro* cultivation protocols have been developed for the propagation and preservation of this plant (Cartabia et al., 2022). In addition, hydroponic cultivation of medicinal plants has attracted the attention of the scientific community and industrial sector, as it can effectively meet the nutrients needs of the plants under controlled/stable environmental conditions (Gontier et al., 2002; Sgherri et al., 2010). Interestingly, these cultivation systems are adapted to the growth of microorganisms such as arbuscular mycorrhizal fungi (AMF) (IJdo et al., 2011; Garcés-Ruiz et al., 2017). These obligate plant symbionts, belonging to the Glomeromycota phylum, provide their hosts with nutrients (especially N and P) in exchange for carbon and lipids (Smith and Read, 2008; Chen et al., 2018). A number of studies have reported their impact on the production of secondary metabolites (SMs) in leaves, roots or fruits/tubers of different crops used as food or for medicinal purposes (Zeng et al., 2013; Avio et al., 2018; Pandey et al., 2018; Kaur and Suseela, 2020; Zhao et al., 2022). For instance, artemisinin in leaves of *Artemisia annua* L. (Chaudhary et al., 2008), caffeic acid and rosmarinic acid in shoots of *Ocimum basilicum* L. (Toussaint et al., 2007), and triterpenes and phenolics in *Dioscorea* spp. (Lu et al., 2015) were increased in AMF-colonized plants. In addition, the AMF *Rhizophagus irregularis* MUCL 41833 was shown to modulate the primary and secondary metabolites (PMs and SMs, respectively) production of *Anchusa officinalis* L. (i.e., shoot and root tissues as well as exudates in the nutrient solution), another important Boraginaceae plant, under a semi-hydroponic (S-H) cultivation system (Cartabia et al., 2021). A similar study, conducted with *A. officinalis* associated with different AMF species (*R. irregularis* MUCL 41833, *Rhizophagus intraradices* MUCL 49410, *Rhizophagus clarus* MUCL 46238, and *R. aggregatus* MUCL 49408), demonstrated that specific symbiotic associations can affect the production of bioactive compounds differently in the same host (Tsiokanos et al., 2022).

Biosynthesis of A/Sd involves the phenylpropanoid and mevalonate pathways (Song et al., 2020). Several genes, responsible for encoding enzymes involved in the biosynthesis of shikonin, have been identified in Boraginaceae plant species (Wang et al., 2014; Wu et al., 2017; Takanashi et al., 2019). The *p*-hydroxybenzoate geranyltransferase (*PGT*) gene plays a key role in coding the enzyme catalyzing the coupling of *p*-hydroxybenzoic acid and geranyl diphosphate to produce *m*-geranyl-*p*-hydroxybenzoic acid, which is the first step in the formation of the basic carbon skeleton leading to A/Sd, and it has been cloned and characterized in cell cultures of *L. erythrorhizon* and *Arnebia euchroma* (Royle) Johnston (Yazaki et al., 2002; Singh et al., 2010). In addition, geranylhydroquinone 3″-hydroxylase (*GHQH*), an enzyme hydroxylating the isoprenoid side chain of geranylhydroquinone (GHQ), a well-known precursor of shikonin, has also been identified in *L. erythrorhizon* cell suspension cultures (Yamamoto et al., 2000). However, to our knowledge, no study has investigated the gene regulation of the biosynthesis of A/Sd in *A. tinctoria*, in particular in the presence of AMF.

In this study, the main objectives were to determine whether (1) AMF from international collection (i.e., GINCO) could enhance the production of A/Sd and whether this effect is more/less significant compared to AMF isolated from *A. tinctoria* grown in the wild; (2) AMF can modulate the expression of genes involved in the A/Sd biosynthetic pathway; (3) the production of A/Sd is affected similarly under semi-hydroponic and pot cultivation systems. To address these objectives, the quantification of A/Sd was performed through High-Performance Liquid Chromatography coupled with Photodiode Array (HPLC-PDA) detection and the related genes expression through Real-Time Quantitative Reverse Transcription PCR (RT-qPCR). Moreover, identification of the main A/Sd was performed using HPLC coupled with High-Resolution Mass Spectrometry detection (HPLC-HRMS/MS).

## 2. Materials and methods

### 2.1. Chemicals

All used organic solvents [i.e., n-hexane 97%, methanol, trifluoroacetic acid (TFA), and acetonitrile (ACN) (VWR INTERNATIONAL, Leuven, Belgium)] were HPLC/LC-MS grade. Water was purified and demineralized with a Milli-Q system manufactured by Millipore (Bedford, MA, USA). Shikonin (purity >98%) was purchased from Cayman Chemical Company (Biomol GmbH, Hamburg, Germany) and used as internal standard.

For gene expression analysis (i.e., RNA extraction) all non-disposable materials (e.g., glass materials, mortars, and pestles) were first treated with RNase AWAY™ Surface Decontaminant (Thermo Fisher Scientific™, Belgium) and 0.1% diethylpyrocarbonate (DEPC)-treated sterile water. All the reagents were prepared with sterilized (121°C for 15 min) DEPC-treated water. Reagents used in RNA extraction were the following: extraction buffer [100 mM Tris–HCl (pH 8.0), 20 mM EDTA, 1.4 M NaCl, 2% CTAB (w/v) and 2% PVP (w/v)], 2% β-mercaptoethanol (v/v), 5 M NaCl, 1.2 M NaCl, 0.38 M trisodium citrate dihydrate, chloroform, isopropanol, and 70% ethanol (stored at −20°C).

### 2.2. Biological materials

*Rhizophagus irregularis* (Błaszk, Wubet, Renker and Buscot) C. Walker and A. Schüßler as [“irregulare”] MUCL 41833 and *Rhizophagus aggregatus* (N.C. Schenck & G.S. Sm.) C. Walker MUCL 49408 were supplied by the *Glomeromycota in vitro* collection (GINCO).^1^ Both AMF were mass-produced on *Zea mays* L. cv. ES Ballade (Euralis, Lescar, France) in 10 L plastic boxes containing sterilized (121°C for 15 min) lava (DCM, Grobbendonk, Belgium). Two other AMF strains (*R. irregularis* and *Septoglomus viscosum*) were isolated from *A. tinctoria* growing in a suburban pine forest, altitude 50 m of Northern Greece (Evaggelistria, Seih Sou, Thessaloniki, special collection permit obtained by the Institute of Plant Breeding and Genetic Resources, Hellenic Agricultural Organization Demeter – IPBGR, HAO Demeter). They were first trapped on *Plantago lanceolata* L. (Ecosem, Belgium) and *Medicago truncatula* Gaertn. (SARDI, Australia) to stimulate the production of numerous spores and then grown as monospores on *P. lanceolata* at the GINCO premises to obtain single species cultures (see section **Isolation and mono-species culture of AMF** in Supplementary material, Supplementary Figures 1–4, and Supplementary Table 1). They were finally mass-produced on *Z. mays* L. in 5 L plastic boxes containing the same sterilized lava substrate as above.

The four AMF strains were grown under greenhouse conditions at 25/18°C (day/night), a relative humidity (RH) of 38%, a photoperiod of 16 h day^–1^ and a photosynthetic photon flux (PPF) of 120 μmol m^–2^ s^–1^.

*Alkanna tinctoria* unrooted *in vitro* explants (International Plant Exchange Network – IPEN – accession number GR-1-BBGK-17,5975) were provide by IPBGR, HAO Demeter (Thessaloniki, Greece). The plants were proliferated on Murashige-Skoog (MS) basal medium enriched with plant growth regulators (PGRs) and rooted on Root Culture 1 (RC1) modified medium free of ammonium nitrate (Cartabia et al., 2022). The rooted *A. tinctoria* plants were further acclimatized following different steps resulting in an optimal plant survival rate (see section “*Alkanna tinctoria* acclimatization protocol” in Supplementary material and Supplementary Figure 5). The acclimatization protocol was applied for all the experiments in order to have plants of the same age and established under identical growth conditions (first acclimatization from 2nd November 2020 to 7th December 2020 and second from 1st February 2021 to 10th March 2021).

### 2.3. Colonization of *A. tinctoria*

After the acclimatization phase, the plants were carefully removed from the substrate, gently washed under a stream of demineralized water, and transplanted into 1 L pots (11 × 11 × 12 cm) containing a sterilized (121°C for 15 min) substrate mixture (2 peatmoss/2 compost/1 perlite/1 quartz 0.4–0.8 mm/1 quartz 1–2 mm) (see Supplementary Figure 5). Total fresh weight (TFW) of the plants was evaluated before AMF inoculation.

In each experiment, plants were associated [i.e., the mycorrhizal (M) treatments] or not [i.e., the non-mycorrhizal (NM) treatments] with AMF. For the M plants, the substrate was half mixed with the AMF-inoculum substrate above, whereas for the NM treatments, the substrate was half mixed with the same AMF-inoculum substrate above but sterilized (121°C for 15 min). The plants were grown in the substrates for two months under greenhouse conditions set at 24/22°C (day/night), RH of 50%, photoperiod of 16 h day^–1^ and LED light (lumigrow) intensity of about 220 μmol m^–2^ s^–1^.

### 2.4. Experiment 1: A/Sd production and genes expression of *A. tinctoria* associated with *R. irregularis* before and after 9 and 37 days in an S-H cultivation system

In Experiment 1, the objective was to evaluate the production of A/Sd and the expression of genes involved in the biosynthetic pathways of A/Sd in *A. tinctoria* roots colonized (later abbreviated for fluency as M^irr^) or not (NM) with *R. irregularis* MUCL 41833, before (T0) and after 9 (T1) and 37 days (T2) of growth in the S-H cultivation system. These harvesting times were based on the study of Cartabia et al. (2021), conducted with *Anchusa officinalis* (another Boraginaceae). The authors made a kinetic study (before the transfer of the plants to the S-H cultivation system and after 9 and 30 days of growth in the S-H cultivation system) on the effect of *R. irregularis* MUCL 41833 on the metabolites profile of *A. officinalis.* Interesting results were obtained at these harvesting times, and therefore were considered in the present study.

Two-month-old M^irr^ and NM plants were gently removed from the pots above and their roots cleaned with demineralized water to eliminate substrate debris (see Supplementary Figure 5). Six plants belonging to the M^irr^ and NM treatments were randomly harvested, and AMF root colonization assessed before (T0) transfer to the S-H cultivation system (see section “2.7. Plants biomass and AMF root colonization”). Fourteen additional plants from the M^irr^ and NM treatments were transferred to the S-H cultivation system as detailed in Cartabia et al. (2021). The plants were placed in 500 ml plastic bottles (VWR INTERNATIONAL, Leuven, Belgium), cut at the base and covered with a 100 μm size pore nylon mesh (Prosep B.V.B.A., Zaventem, Belgium) glued on the top. The bottles (called containers thereafter) were used bottom-up, filled with 32 g of perlite (KNAUF GMBH, Dortmund, Germany), covered with a superficial layer of black lava rock (1–3 mm) and wrapped in aluminum foil to avoid algae development and inhibition of shikonin production (as shikonin is sensitive to light) (Yazaki, 2017). The containers were transferred randomly in holes made in flex foam supports and were maintained in the greenhouse set at the same conditions as described above. A 90% P-impoverished modified Hoagland solution (see Garcés-Ruiz et al., 2017; and Supplementary Table 2) lacking ammonium nitrate (NH_4_NO_3_) [as NH_4_^+^ is an inhibitor of shikonin production (Yazaki, 2017)] was used at two different concentrations as detailed in Cartabia et al. (2021). After 7 days of acclimatization, the circulatory system was started. Each container was connected to a 1 L glass bottle covered with aluminum foil containing Hoagland^dil100X^ solution. A 4.6 mm diameter black supply pipe (GARDENA^®^, Micro-Drip System, Ulm, Germany) connected a dropper cap fixed on the bottom of the plant container with the glass bottle, and another black pipe of the same diameter connected the glass bottle to the upper part of the plant container via a multichannel peristaltic pump (Gilson’s Minipuls Evolution, France). Once the circulation started, the nutrient solution was pumped from the glass bottle into the plant container and the liquid flowed back by gravity into the bottle. Before starting with the circulation, initial flushing was performed as described in Cartabia et al. (2021). Then, four successive circulations were performed at a velocity of 7.5 ml min^–1^ for different durations: 42 h at day 9 (T1), 8 h at day 17, 24, and 31, and finally 42 h at day 37 (T2).

### 2.5. Experiment 2: A/Sd production and genes expression of *A. tinctoria* associated with different AMF strains after 9 days in an S-H cultivation system

In Experiment 2, the objective was to evaluate the production of A/Sd and the expression of genes involved in the biosynthetic pathways of A/Sd in *A. tinctoria* roots colonized (later abbreviated for fluency as M^irr^, M^aggreg^, M^Rhiz^, and M^Sept^) or not (NM) with *R. irregularis* MUCL 41833, *R. aggregatus* MUCL 49408, the native *R. irregularis* and *S. viscosum* and grown for 9 days in the S-H cultivation system. This harvesting time was based on the study of Tsiokanos et al. (2022) where different AMF strains were associated to *A. officinalis*, and plants were harvested after 9 days of growth in the S-H cultivation system. Interesting results were obtained following this harvesting time and therefore considered in the present study. The same procedure as in Experiment 1 was applied. Briefly, after cleaning the roots system, 2-month-old plants (six per treatment) were transferred in the S-H cultivation system, and the containers were randomly placed in the holes made in the flex foam supports. Then, after the acclimatization period of 7 days, a regular circulation was initiated and maintained at 7.5 ml min^–1^ for 42 h.

### 2.6. Experiment 3: A/Sd production and genes expression of *A. tinctoria* associated with different AMF strains in an 85 days pot-experiment in the greenhouse

In Experiment 3, the objective was to evaluate the production of A/Sd and the expression of genes involved in the biosynthetic pathways of A/Sd in *A. tinctoria* roots colonized (M^irr^, M^Rhiz^, and M^Sept^) or not (NM) with *R. irregularis* MUCL 41833, the native *R. irregularis* and *S. viscosum* and growing for 85 days in pots under greenhouse conditions. In this Experiment, *R. aggregatus* MUCL 49408 was not considered due to inoculum limitation. Six plants per treatment were gently removed from the 1 L pots above and transplanted in 3 L pots (3 × 18.5 × 17 cm) containing a sterilized (2 × 121°C for 15 min) substrate mixture (2 peatmoss/2 compost/1 perlite/1 quartz 0.4–0.8 mm/1 quartz 1–2 mm). They received 200 ml Hoagland^dil200X^ solution and were randomly moved every week until harvest. The plants were grown under the same conditions as for the S-H cultivation system in Experiments 1 and 2.

### 2.7. Plants biomass and AMF root colonization

For the three experiments, plants were harvested and biomass as well as AMF root colonization evaluated. In Experiment 1, plants were harvested before transfer to the S-H cultivation system (T0) and after 9 (T1) and 37 days (T2) corresponding to 57 and 94 days after AMF inoculation of the plants, respectively. In Experiment 2, they were harvested after 9 days (i.e., 79 days after AMF inoculation of the plants), and in Experiment 3, 85 days after AMF inoculation of the plants.

Whatever the experiment, shoots were separated from roots (cleaned under demineralized water and gently dried). Shoot fresh weigh (SFW) and roots fresh weight (RFW) were then measured. Root colonization was further evaluated by McGonigle et al. (1990). Roots (an approximate of 2 g fresh material) were cut into circa 1 cm length pieces and placed in 50-ml Falcon tubes. Twenty-five milliliter of KOH 10% was added to the roots before incubation at 70°C for 40 min. The KOH solution was then removed and replaced with H_2_O_2_ 3.5% before incubation at 70°C for 5 min. The roots were then rinsed with demineralized water and HCl 1% was added for 1 min at room temperature. After discarding the solution, the roots were stained with ink 2% (Parker Blue Ink, United States) in HCl 1% (Walker, 2005) by placing the tubes at 70°C for 30 min. The roots were finally rinsed with demineralized water and kept in lactoglycerol (lactic acid/glycerol/distilled water, 1:1:1, v/v) until observation. For colonization assessment, the root fragments were placed on microscope slides and covered with 40 × 22 mm coverslips before observation under a bright field light microscope (Olympus BH2-RFCA, Japan) at ×10 magnification. Around 200 root intersections were observed for each plant in Experiments 1 and 2 and around 300 intersections for the plants in Experiment 3. The total colonization percentage (TC%) of roots (e.g., hyphae, arbuscules, and vesicles/spores), and arbuscules colonization percentage (AC%) was further calculated (McGonigle et al., 1990).

The remaining root system of each replicate was divided in two parts of around 5 g each for metabolites and gene expression analysis. Regarding the analysis of metabolites, roots were freeze-dried during 72 h and kept at −80°C before proceeding with A/Sd extraction (see section “2.8. Quantitative and qualitative analysis of A/Sd in *A. tinctoria* roots”). For analysis of gene expression, roots were transferred within 5–10 min after sampling into liquid nitrogen and kept at −80°C before proceeding with the RNA extraction (see section “2.9. Analysis of A/Sd target genes expression in *A. tinctoria* roots”).

### 2.8. Quantitative and qualitative analysis of A/Sd in *A. tinctoria* roots

#### 2.8.1. Standards stock solutions, calibration curves, and validation parameters

The standard stock solution of shikonin was serially diluted in HPLC-grade methanol to obtain a range of concentrations from 0.05 to 0.8 mg ml^–1^ (i.e., stock solution of 1 mg ml^–1^ of shikonin was prepared and diluted in HPLC-grade methanol to obtain the calibration solutions of 0.8, 0.6, 0.4, 0.2, 0.1, and 0.05 mg ml^–1^). The calibration curve was made by plotting the average peak areas of three independent experiments versus the concentration of each analyte. The method was validated with three independent series of experiments based on total error and tolerance intervals (Hubert et al., 2003). Specificity was evaluated by analysis of an extract sample by HPLC-HRMS and comparison of MS^1^ signals of blank, shikonin standard, and *A. tinctoria* extract at 0.8 μg ml^–1^, respectively (see section “2.8.4. MS data treatment, organization, and dereplication”). Linear regression equation, response function, linearity (*R*^2^), precision, trueness, accuracy, limit of detection (LOD), limit of quantification (LOQ), and stability were provided (see section “HPLC quantification: methodology validation” in Supplementary material, Supplementary Figures 6, 7, and Supplementary Table 3).

#### 2.8.2. Samples preparation

For the three experiments, primary and secondary roots of *A. tinctoria* plants were freeze-dried separately and reduced into powder using liquid nitrogen, mortar and pestle. Fifty milligram of each root type was then mixed in glass test tube (labbox 10 ml neutral glass, TU04-160-100) closed with a plastic cap. Then, the total 100 mg of root material was subjected to a 20-min ultrasound-assisted extraction (two cycles), using *n*-hexane 97% (2 ml) at room temperature. The samples were finally centrifuged at 1,500 rpm for 10 min at 4°C and the supernatants of each cycle were combined and evaporated at room temperature. The extraction protocol was adapted from Bossard et al. (2022).

Prior to analysis, the plant extracts and nutrient solution residues were weighed and solubilized in methanol and were filtered through a 45-μm PTFE membrane (Whatman™, Maidstone, UK). Each sample was adjusted to the final concentration of 2 mg ml^–1^, using methanol LCMS-grade.

#### 2.8.3. HPLC-PDA and HPLC-HRMS/MS analysis

For the three experiments, the quantification of HNQ enantiomers (A/Sd) in their free form (not linked to sugars) was performed following the protocol of Bossard et al. (2022). For HPLC-PDA analysis, we used an Accela HPLC system (Thermo Fischer Scientific™, Bremen, Germany) coupled with a photodiode array (PDA) detector, an autosampler equipped with a conventional sample tray compartment with its cooler (set at 4°C), an injection system with a sample loop of 20 μl, and a quaternary pump, all piloted by ChromQuest software. The column used was an Alltech ALLTIME C8 250 × 4.6 mm, packed with 5 μm particles. Twenty microliter of sample was injected in full loop injection mode by the autosampler. The column was eluted at constant flow rate of 1 ml min^–1^ using a binary solvent system: solvent A, Milli-Q water 0.1% TFA and solvent B, ACN, isocratic mode (25% A: 75% B). Quantification analyses were conducted at a wavelength of 510 nm corresponding to the maximum absorption of A/Sd and their content was calculated according to the shikonin standard curve. The total shikonin equivalent production was calculated as the sum of peaks corresponding to A/Sd free forms (i.e., by integration of the different peaks identified as A/Sd-type compounds by their UV spectra) contained in the root powder, reported as mg g^–1^ root powder using the same shikonin standard curve (so not corresponding to an absolute quantification of each compound). Peaks were identified by comparing their retention times (RT) and UV spectra with the standard chromatogram of shikonin. The chromatographic profiles were in agreement with the chemical profile reported by Bossard et al. (2022), for A/Sd obtained from *A. tinctoria* roots. In total, 18 M and 18 NM root samples (6 NM and 6 M^irr^ plants for each time harvest-T0, T1, and T2) were considered in Experiment 1, 30 root samples (6 M^irr^, 6 M^aggreg^, 6 M^Rhiz^, 6 M^Sept^, and 6 NM) in Experiment 2, and 24 root samples (6 M^irr^, 6 M^Rhiz^, 6 M^Sept^, and 6 NM) in Experiment 3. All samples were analyzed in duplicates.

The HPLC-HRMS/MS analysis was performed at the end of Experiments 2 and 3 on an HPLC-PDA-HRMS system consisting of an Accela pump and PDA detector (Thermo Fisher Scientific™, Bremen, Germany) connected with a LTQ orbitrap XL mass spectrometer (Thermo Fisher Scientific™, Bremen, Germany). The instrument was controlled using a Thermo Fisher Scientific™ Xcalibur X software. The LC separation was done as reported above except for the solvent A: Milli-Q water 0.1% formic acid (FA). The chromatograms were recorded between 200 and 600 nm. HRMS analyses were realized in ESI positive and negative modes with the following inlet conditions for the positive mode: capillary temperature 250°C; sheath gas flow 10 a.u.; auxiliary gas flow 5 a.u. and sweep gas flow 5 a.u; ionization spray voltage 3 kV; capillary voltage of 15 V; tube lens voltage of 90 V. For negative mode, the only differences from the positive mode were the capillary voltage (−10 V) and tube lens voltage (−125 V). The data-dependent MS/MS events were performed on the three most intense ions detected in full scans MS. Peaks were tentatively identified by comparing their HRMS and MS/MS spectra with the literature data of A/Sd.

#### 2.8.4. MS data treatment, organization, and dereplication

All HRMS run data (.RAW) files were treated using MZmine software suite version 2.5.3 (Pluskal et al., 2010). For mass detection at MS^1^ level, the noise level was set to 1.5 × 10^4^ for positive mode and to 8.5 × 10^3^ for negative mode. For MS^2^ detection, the noise level was set to 1. The ADAP chromatogram builder was used and set to a minimum group size of scans of 4, a minimum group intensity of 1.0 × 10^4^, a minimum highest intensity of 1.0 × 10^4^, and *m/z* tolerance of 8 ppm. The ADAP algorithm (wavelets) was used for chromatogram deconvolution. The intensity window signal to noise (S/N) was used as a S/N estimator with S/N ratio set at 10, a minimum feature height of 1.0 × 10^4^, a coefficient area threshold of 25, a peak duration ranging from 0.02 to 0.8 min, and a RT wavelet range from 0.02 to 0.2 min. Isotopes were detected using the isotope peak grouper with a *m/z* tolerance of 8 ppm, a RT tolerance of 0.02 min (absolute), a maximum charge set at 1, and the representative isotope used was the most intense. Then, the aligned list peak was gap-filled with RT range of 0.05 min and *m/z* tolerance of 8 ppm. The resulting list was filtered using the peak list rows filter option to remove all the duplicates and all the features without MS^2^ spectrum associated.

A molecular network was constructed from the .mgf file exported from MZmine, using the online workflow on the GNPS website (Wang et al., 2016). The precursor ion mass tolerance was set to 0.02 Da with a MS/MS fragment ion tolerance of 0.02 Da. A network was then created where edges were filtered to have a cosine of 0.7 and more than 3 matched peaks. The spectra in the network were then searched against GNPS’s spectral libraries filtered under the same conditions as before. Putative identification was carried out comparing available MS/MS fragmentation patterns from the literature. Data visualization was achieved using Cytoscape 3.8.0 (Shannon et al., 2003). Peak area data from the .csv file obtained from MZmine was added to the network. Size nodes were set proportionally to the total area of each peak detected in both analyzed extracts.

### 2.9. Analysis of A/Sd target genes expression in *A. tinctoria* roots

#### 2.9.1. Total RNA extraction

Frozen roots of each replicate were ground in liquid nitrogen (−196°C) with a pestle and mortar. Total RNA extraction was done on 0.2 g root material using the protocol from Xu et al. (2010) slightly modified. Main differences were in the extraction buffer composition and in the last steps of the protocol, where the pellet was washed with 70% ethanol and the total RNA was dissolved in 55 μl DEPC-treated water. Further, the total RNA was treated with TURBO DNA-free™ Kit (Thermo Fisher Scientific™, Belgium), according to the manufacturer protocol. The RNA of all samples was loaded on 1.5% agarose gel, electrophoresed to separate RNA, stained with GelRed^®^ (Biotium, United States), and visualized under UV light to assess the integrity of ribosomal bands. Moreover, concentration of each RNA sample was measured using NanoDrop-ND 1000 UV-vis Spectrophotometer (NanoDrop^®^ Technologies, United States) and RNA purity estimated from the A260/A280 and A260/A230 absorbance ratios. Finally, a 1 μg aliquot of total RNA was used for the first-strand cDNA synthesis according to the protocol of the Transcriptor High Fidelity cDNA Synthesis Kit (Roche, Montreal, QC, Canada). For each RNA sample, a reaction without Transcriptor High Fidelity Reverse Transcriptase (Hifi RT) enzyme was performed as a control for contamination by genomic DNA.

Five biological replicates (i.e., means of relative genes expression) for M and NM treatments at each time harvest (T0, T1, and T2) were considered in Experiment 1, six biological replicates for treatments M^irr^, M^aggreg^, M^Rhiz^, M^Sept^, and NM in Experiment 2, and six biological replicates for treatments M^irr^, M^Rhiz^, and NM and five for M^Sept^ in Experiment 3.

For all the experiments, tissue samples for A/Sd and molecular determinations were simultaneously collected at the same period of the day, between 9:00 and 11:00 a.m.

#### 2.9.2. Real-time quantitative PCR

For RT-qPCR, the expression of three target genes: *LePGT1*, *LePGT2*, and *GHQH* involved in the shikonin (A/Sd) biosynthesis was analyzed (see section “Analysis of A/Sd target genes expression in *A. tinctoria* roots” in Supplementary material and Supplementary Table 4). Since no published genomic resources are available for *A. tinctoria*, a previously published primer for amplification of *LePGT1* in *Onosma paniculatum* Bur. et Franch was used, while for *PGT2* and *GHQH* new primer pairs were designed. Sequences of each gene (*LePGT2* or *GHQH*) from two phylogenetically distant Boraginaceae species (*L. erythrorhizon* and *A. euchroma*) were obtained from NCBI, aligned, and primers generated from conserved regions (see section “Alignments used to design primers” in Supplementary material). The glyceraldehyde-3-phosphate dehydrogenase gene (*GAPDH*) was used as internal reference control. RT-qPCR was performed using a LightCycler^®^ FastStart Essential DNA Green Master (Roche) in 10 μl volume of reaction formed as follows: 5 μl Master mix (or Mix SYBR 2x), 0.5 μl of each primer from the pair (10 μM), and 4 μl cDNA (dil 5x). The reaction was carried out in a Roche LightCycler^®^ 96 System using the following parameters: 10 min at 95°C, followed by 40 (for housekeeping gene) and 50 (for target genes) cycles of denaturation (95°C, 10 s)/annealing (60°C housekeeping gene/56°C target genes, 15 s)/extension (72°C, 10 s), and finalized by a standard melting curve analysis (95°C). Reactions were performed in three replicates. Normalization was achieved for each experiment separately using the reference gene (i.e., *GAPDH*) and the “Pfaffl” method (Pfaffl, 2001).

### 2.10. Statistical analysis

For all the experiments, a one-way ANOVA followed by HSD Tukey *post-hoc* test (*p* < 0.05) was applied to discriminate between means for plant growth parameters (i.e., SFW, RFW, and TFW), AMF colonization (i.e., TC% and AC%), A/Sd content, and relative genes expression at different time points (Experiment 1) or different AMF treatments (Experiments 2 and 3). Moreover, in Experiment 1, differences between M and NM treatments were highlighted by pairwise comparison with Bonferroni correction (*p* < 0.05) at each harvesting time (T0, T1, and T2). For all parameters, normal distribution of residuals variance and normality was checked before analyses. Non-normal data were normalized by log10 transformation before analysis. Data analyses were performed by IBM SPSS Statistics for Windows, version 28 (IBM Corp., Armonk, N.Y., USA).

## 3. Results

### 3.1. Plants biomass and root colonization by AMF

In Experiment 1, no significant differences between harvesting times (before plants transfer to the containers-T0, and after 9-T1 and 37-T2 days of growth in the S-H cultivation system) were noticed for SFW, RFW, and TFW of plants in the M^irr^ treatment and for SFW of plants in the NM treatment. Conversely, a significantly greater RFW was noticed at T1 and T2 as compared to T0, and greater TFW at T2 compared to T0 for plants in the NM treatment. Irrespective of the harvest time, no significant difference was reported between the SFW of plants in the M^irr^ and NM treatments, while at T2, the RFW and TFW of the plants in the NM treatment was significantly greater than that in the M^irr^ treatment. In the M^irr^ treatment, a significant decrease in TC% was observed at T2 compared to T0 and T1 and in AC% at T1 and T2 compared to T0 (Table 1). No root colonization was observed in plants of the NM treatment.

**TABLE 1:** Shoot, root and total fresh weights (SFW, RFW, and TFW, respectively), total colonization percentage (TC%), arbuscules colonization percentage (AC%), and total A/Sd expressed as shikonin equivalent content of *A. tinctoria* plants inoculated (M^irr^) or not (NM) with *R. irregularis* MUCL 41833 before (T0) and after 9 (T1) and 37 (T2) days in the S-H cultivation system (Experiment 1), and of *A. tinctoria* plants inoculated (M^irr^, M^aggreg^, M^Rhiz^, M^Sept^) or not (NM) with different AMF strains (two from GINCO – *R. irregularis* MUCL 41833 and *R. aggregatus* MUCL 49408, and two isolated from wild *A. tinctoria* – *R. irregularis* and *S. viscosum*) after 9 days in the S-H cultivation system (Experiment 2).

Experiment 1							
Treatments	Time	SFW (g)	RFW (g)	TFW (g)	TC%	AC%	Total shikonin equivalent mg g^–1^ root powder
M^irr^	T0	14 ± 2.3	10 ± 1.6	24 ± 2.4	61 ± 7 a	34 ± 4 a	3.3 ± 1.5
	T1	13 ± 2.7	10.8 ± 2.2	23.7 ± 3.6	53 ± 7 a	17 ± 5 b	1.9 ± 1.5
	T2	13 ± 1.2	12.4 ± 1.1*	25.7 ± 2*	29 ± 4 b	12 ± 3 b	2.1 ± 1
NM	T0	13.5 ± 2	7.9 ± 2.3 b	21.4 ± 3.8 b	–	–	2.5 ± 1
	T1	14.2 ± 2	11 ± 2.4 a	25.2 ± 3.7 ab	–	–	2.6 ± 1.1
	T2	13.6 ± 0.6	14 ± 1 a*	28 ± 1 a*	–	–	2.3 ± 0.7
**Experiment 2**							
**Treatments**	**SFW (g)**	**RFW (g)**	**TFW (g)**	**TC%**	**AC%**	**Total shikonin equivalent mg g^–1^ root powder**	
M^Rhiz^	9.8 ± 1	11.3 ± 1	21 ± 1.8	44 ± 7	21 ± 5 a	8.7 ± 1.3 a	
M^Sept^	11.1 ± 2	13.1 ± 2.6	24.2 ± 1	39 ± 9	17 ± 7 ab	6.8 ± 2.2 ab	
M^irr^	11.2 ± 1	12.8 ± 1.7	24.2 ± 2.4	42 ± 6	16 ± 2 ab	4.3 ± 2.1 b	
M^aggreg^	10.3 ± 5	11 ± 3.3	20 ± 5	43 ± 9	12 ± 4 b	5 ± 0.6 b	
NM	10.3 ± 2	11 ± 2.1	21.5 ± 3.3	–	–	4.9 ± 1.9 b	

The parameters measured are expressed as mean ± standard deviation (SD) of five replicates per treatment (M^irr^ and NM) and harvesting time (T0, T1, and T2) in Experiment 1, and 6 replicates per treatment (M^irr^, M^aggreg^, M^Rhiz^, M^Sept^, and NM) in Experiment 2. Means followed by different lowercase letters within the same column are significantly different according to HSD Tukey *post-hoc* test (*p* < 0.05) (Experiments 1 and 2). Means followed by asterisk within the same column are significantly different according to pairwise comparison with Bonferroni correction (*p* < 0.05) (Experiment 1).

In Experiment 2, no significant differences were observed in SFW, RFW and TFW and in TC% between the different treatments (M^irr^, M^aggreg^, M^Rhiz^, M^Sept^, and NM) after 9 days of growth of the plants in the S-H cultivation system. Conversely, a significant higher AC% was observed for the plants in the M^Rhiz^ treatment compared to those in the M^aggreg^ treatment, while intermediate values were noticed in the plants of the M^Sept^ and M^irr^ treatments (Table 1). No root colonization was observed in plants of the NM treatment.

In Experiment 3, no significant differences were observed in SFW and RFW of plants between the different treatments (M^irr^, M^Rhiz^, M^Sept^, and NM) after 85 days of growth in pots. Similarly, no significant differences were observed for TC% between the different treatments, while a significantly higher AC% was observed for the plants in the M^Sept^ treatment compared to those of the M*^irr^* treatment. Plants in the M^Rhiz^ treatment had intermediate value (Table 2). No root colonization was observed in plants of the NM treatment.

**TABLE 2:** Shoot and root fresh weights (SFW and RFW, respectively), total colonization percentage (TC%), arbuscules colonization percentage (AC%), shikonin, and total A/Sd expressed as shikonin equivalent of *A. tinctoria* inoculated (M^irr^, M^Rhiz^, and M^Sept^) or not (NM) with different AMF strains (one from GINCO – *R. irregularis* MUCL 41833, and two isolated from wild *A. tinctoria* – *R. irregularis* and *S. viscosum*) after 85 days in pots under greenhouse conditions (Experiment 3).

Treatments	SFW (g)	RFW (g)	TC%	AC%	mg shikonin g^–1^ root powder	Total shikonin equivalent mg g^–1^ root powder
M^irr^	15.8 ± 2	13.3 ± 2.6	25 ± 6.6	4 ± 2 b	0.04 ± 0.01 b	7.2 ± 1 b
M^Rhiz^	17.7 ± 0.6	14 ± 2.1	23 ± 16	8 ± 10 ab	0.12 ± 0.07 a	9.6 ± 0.7 a
M^Sept^	16.3 ± 2.3	12.8 ± 1.7	25 ± 13	22 ± 11 a	0.06 ± 0.02 b	7.8 ± 1 b
NM	16.1 ± 1.3	14.4 ± 0.6	–	–	0.04 ± 0.01 b	8 ± 1 b

The parameters measured are expressed as mean ± standard deviation (SD) of six replicates per treatment (M^irr^, M^Rhiz^, M^Sept^, and NM). Means followed by different lowercase letters within the same column are significantly different according to HSD Tukey *post-hoc* test (*p* < 0.05).

### 3.2. Alkannin/shikonin and their derivatives content

In Experiment 1, whatever the harvesting time (T0, T1, or T2) or treatment (M^irr^ or NM) no significant differences in content of A/Sd were noticed (Table 1).

In Experiment 2, a significant higher content of total A/Sd expressed as shikonin equivalent was observed in the plants of the M^Rhiz^ treatment compared to those in the M^irr^, M^agreeg^, and NM treatments, while an intermediate value was observed for the plants in the M^Sept^ treatment (Table 1).

In Experiment 3, a significantly higher content of shikonin, whose values were nevertheless below the LOQ (Supplementary Table 2), and in total A/Sd expressed as shikonin equivalent, was reported in the roots of the plants in the M^Rhiz^ treatment compared to those in the M^irr^ and NM treatments, while an intermediate value was observed for the plants in the M^Sept^ treatment (Table 2).

### 3.3. Alkannin/shikonin and their derivatives target genes expression

**FIGURE 1:**
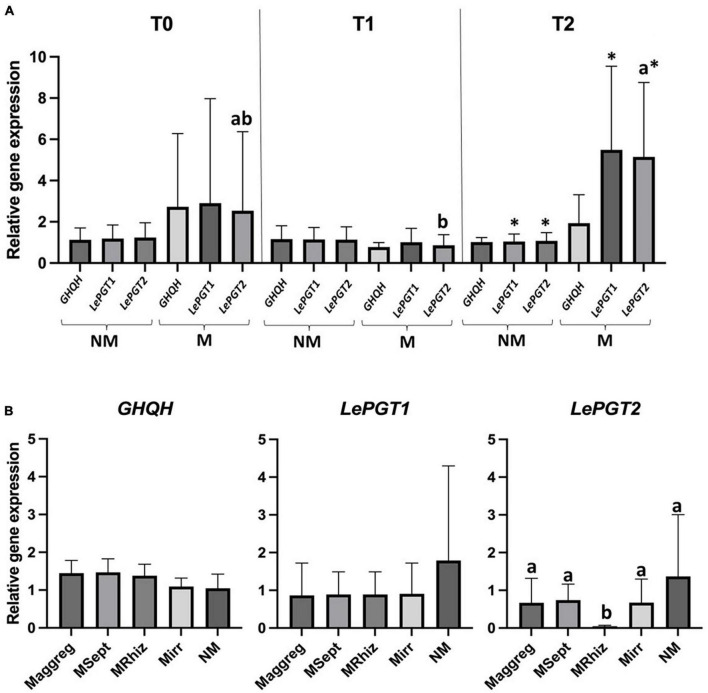
**(A)** Graphical representation of RT-qPCR relative genes expression analysis of *GHQH*, *LePGT1*, and *LePGT2* in *A. tinctoria* roots inoculated (M^irr^) or not (NM) with *R. irregularis* MUCL 41833 before (T0) and after 9 (T1) and 37 (T2) days in the S-H cultivation system (Experiment 1); **(B)** graphical representation of RT-qPCR relative genes expression analysis of *GHQH*, *LePGT1*, and *LePGT2* in *A. tinctoria* roots inoculated (M^irr^, M^aggreg^, M^Rhiz^, and M^Sept^) or not (NM) with different AMF strains (two from GINCO – *R. irregularis* MUCL 41833 and *R. aggregatus* MUCL 49408, and two isolated from wild *A. tinctoria* – *R. irregularis* and *S. viscosum*) after 9 days in the S-H cultivation system (Experiment 2). Means followed by different lowercase letters within the same column are significantly different according to HSD Tukey *post-hoc* test (*p* < 0.05). Means followed by asterisk within the same column are significantly different according to pairwise comparison with Bonferroni correction (*p* < 0.05).

In Experiment 1, whatever the time of evaluation (T0, T1, or T2) or treatment (M^irr^ or NM) no significant differences were noticed in gene expression of *GHQH*. Similarly, no significant differences between harvesting times were observed in expression of *LePGT1* gene of the plants in the M^irr^ treatment and expression of *LePGT1* and *LePGT2* genes of the plants in the NM treatment. Conversely, a significantly higher expression of LePGT2 gene was observed at T2 compared to T1 for the plants in the M^irr^ treatment, while an intermediate gene expression was noticed at T0. A significant higher relative expression of both *LePGT1* and *LePGT2* genes was observed in the plants of the M^irr^ treatment compared to those in the NM treatment at T2 (Figure 1A).

In Experiment 2, whatever the treatment (M^irr^, M^aggreg^, M^Rhiz^, M^Sept^, and NM), no significant differences were observed in expression of *GHQH* and *LePGT1* genes. A significant lower relative expression of *LePGT2* was observed in the plants of the M^Rhiz^ treatment compared with the other treatments that did not differ among them (Figure 1B).

In Experiment 3, *LePGT2* target gene was not considered due to contamination by the primer dimer. Whatever the treatment (M^irr^, M^Rhiz^, M^Sept^, and NM), no significant differences were observed in expression of *GHQH* and *LePGT1* genes (data not presented).

### 3.4. HPLC-HRMS/MS analysis

**FIGURE 2:**
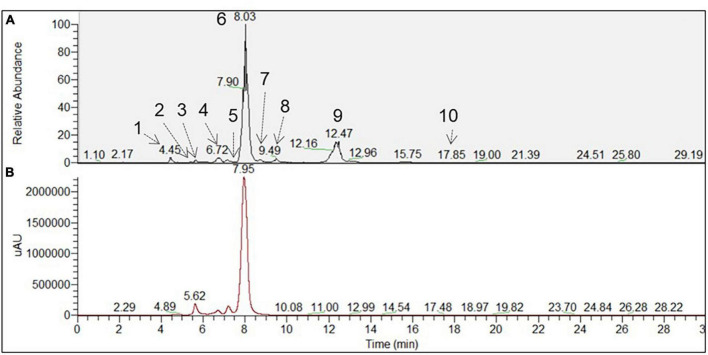
Chromatographic profile of *A. tinctoria* root samples associated with *Septoglomus viscosum* detected under **(A)** HPLC-MS (BP+) and **(B)** HPLC-PDA (510 nm). Each peak defined by a number referred to a tentative identified compound, which has been described in Table 4.

**FIGURE 3:**
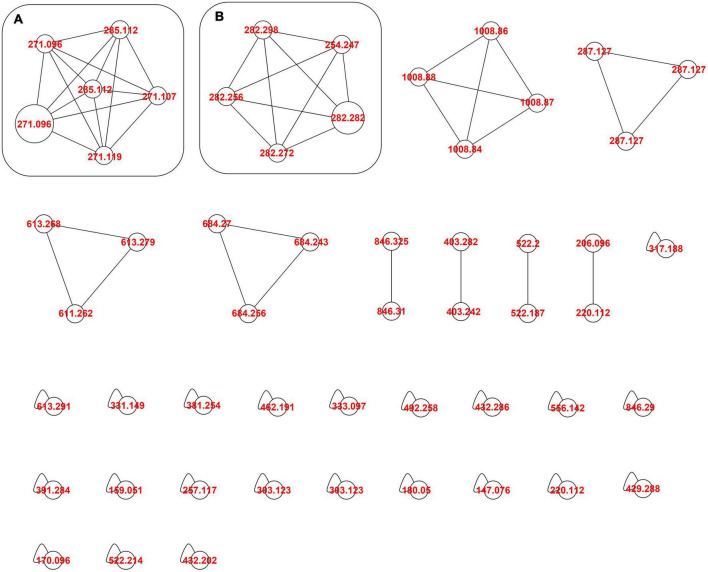
Molecular network of *A. tinctoria* root extracts obtained in Experiment 2 in positive mode. **(A)** HNQ naphthoquinone’s enantiomers (A/Sd); **(B)** lipid amides. Clusters were built with a cosine of 0.7 with a minimum of 3 common ions. Size nodes are proportional to corresponding peak area.

**FIGURE 4:**
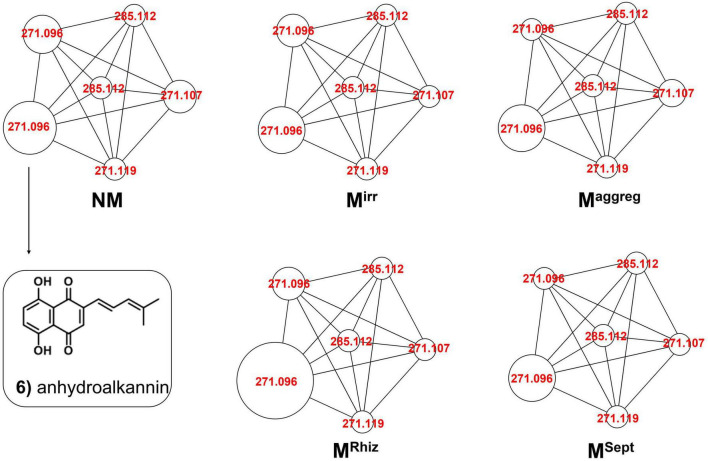
Comparative molecular networking of shikonin derivatives cluster (different nodes) between *A. tinctoria* roots inoculated or not (control) with different AMF strains (two from GINCO – *R. irregularis* MUCL 41833 and *R. aggregatus* MUCL 49408, and two isolated from wild *A. tinctoria* – *R. irregularis* and *S. viscosum*) and growing for 9 days in the S-H cultivation system (Experiment 2).

**FIGURE 5:**
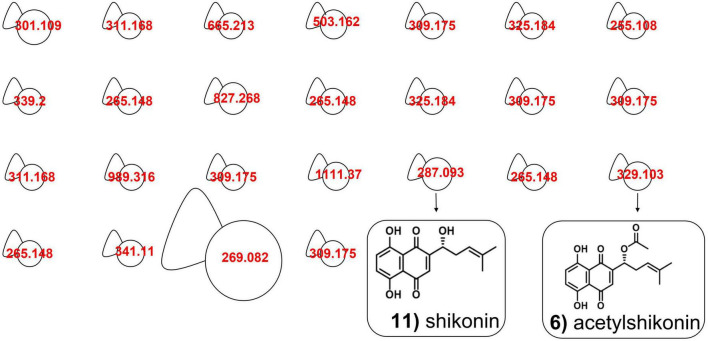
Molecular network in *A. tinctoria* root extracts obtained in Experiment 3 in negative mode. Size nodes are proportional to corresponding peak area.

To better recognize the metabolites produced in *A. tinctoria* roots, the main chemical compounds were tentatively identified in Experiments 2 and 3 by performing a dereplication strategy based on HPLC-HRMS/MS analysis (Figure 2 and Tables 3, 4) and molecular network representation (Figures 3–5). These analyses were not performed in Experiment 1, since no significant differences were reported in the production of A/Sd.

**TABLE 3:** Putative identification of major chemical constituents in *A. tinctoria* extracts from Experiment 2 (positive mode ESI).

	Code	Retention time (min)	UV	(*m/z*)	MS major ion(s)	Molecular formula	Δ ppm	Δ mDa	MS/MS fragments (*m*/*z*)	Putative identification
Cluster A	1	4.60	513, 272	303.1224	[M + H]^+^	C_17_H_18_O_5_	−2.80	−0.85	285.1119 243.1013 233.0442	Methylshikonin isomer I
	2	5.41	n.d.	220.1119	[M + H]^+^	C_16_H_13_N	−3.29	−0.72	205.0883 142.0648 128.0618	*N*-phenyl-naphthylamine
	3	5.66	515, 272, 489	287.1267	[M + H]^+^	C_17_H_18_O_4_	−5.69	−1.63	219.0649 269.1168 231.0648 245.0804	*O*-methyl-1′-deoxyshikonin
	4	6.74	415, 258	285.1125 303.1228	[M + H-H_2_O]^+^ [M + H]^+^	C_17_H_18_O_5_	−0.65	−0.18	267.1012 243.0649 233.0443	Methylshikonin isomer II
	5	7.16	n.d.	403.2317	[M + H]^+^	C_20_H_34_O_8_	−3.70	−1.49	361.2221 329.1594 273.0968 213.0757	Not identified
	6	8.00	517, 484, 281	271.0955 541.1843	[M + H]^+^ [2M + H]^+^	C_16_H_14_O_4_	−5.66	−1.53	229.0493 253.0855 165.0181 191.0338 179.0338 243.1014	Anhydroalkannin
Cluster B	7	8.76	n.d.	254.2475	[M + H]^+^	C_16_H_31_NO	−3.50	−0.89	237.2209 219.2104	Palmitoleamide
	8	9.52	n.d.	280.2627 559.5198	[M + H]^+^ [2M + H]^+^	C_18_H_33_NO	−4.78	−1.34	263.2365 245.2260	Linoleamide
	9	12.33	n.d.	282.2793 563.2793	[M + H]^+^ [2M + H] +	C_18_H_35_NO	−1.38	−0.39	265.2522 247.2417	Oleamide
	10	17.87	n.d.	284.2943	[M + H] +	C_18_H_37_NO	−3.66	−1.04	267.2267	Stearamide

Δm, mass errors; [M − H]^+^, *m/z* of the protonated molecular ion in positive ionization mode; *m/z*, mass to charge ratio.

**TABLE 4:** Putative identification of major chemical constituents in *A. tinctoria* extracts (Experiment 3, negative mode, ESI).

Code	Retention time (min)	UV	(*m/z*)	MS major ion(s)	Molecular formula	Δ ppm	Δ mDa	MS/MS fragments (*m*/*z*)	Putative identification
1	4.60	513, 272.	301.1086	[M − H]^–^	C_17_H_18_O_5_	3.30	1.00	286.0847 232.0375	Methylshikonin isomer I
11	4.78	515, 274, 490	287.0934	[M − H] ^–^	C_16_H_16_O_5_	5.06	1.45	218.0227 190.0273	Shikonin
12	5.26	n.d.	797.2762	[M − H] ^–^	C_33_H_50_O_22_	5.83	4.65	527.1894	Not identified
13	5.85	516, 274, 490	329.1030	[M − H] ^–^	C_18_H_18_O_6_	1.48	0.49	269.0829 251.0709 241.0865	Acetylshikonin
14	7.62	n.d.	1,111.3696	[M − H] ^–^	C_47_H_68_O_30_	−1.90	−2.12	841.2741 571.1847 1023.3120	Not identified
15	7.98	n.d.	1,147.3676	[M − H] ^–^	C_50_H_68_O_30_	−3.59	−4.12	877.2743 607.1854 1047.3101	Not identified
6	8.00	517, 484, 281	269.0829	[M − H] ^–^	C_16_H_14_O_4_	5.63	1.52	251.0712 241.0868	Anhydroalkannin

Δm, mass errors; [M − H] ^–^, *m/z* of the deprotonated molecular in negative ionization mode; *m/z*, mass to charge ratio.

In Experiment 2, the HPLC-HRMS/MS analysis was performed in positive and negative modes. However, shikonin was not detected in any root sample analyzed. Dereplication analyses of shikonin derivatives were done on positive mode to compare our results with published data available in the literature (Bossard et al., 2022). The major detected compounds were organized in two distinctive clusters: A/Sd naphthoquinones (cluster A, Figures 3A and Table 3) and lipid amides derivatives (cluster B, Figure 3B and Table 3). In cluster A, the first eluted derivative was putatively identified as a methylshikonin isomer (1) which gave a protonated molecular ion [M + H]^+^ at *m*/*z* 303 and fragmented to *m*/*z* 285, 243, and 233, corresponding to the loss of a H_2_O molecule, a C_2_H_4_O_2_ and a C_5_H_10_ fragment from the alkyl chain. Similar fragmentation pattern was observed for compound 4 with a protonated dehydrated molecular ion [M + H-H_2_O]^+^, putatively identified as a second methylshikonin derivative. Similar loss was observed for compounds 3 and 6, putatively corresponding to methyl-1′-deoxyshikonin and anhydroalkannin at *m*/*z* of 287 and 271, respectively. Data also indicated that the major shikonin derivative produced by the plants under these conditions was anhydroalkannin (6) (Kyogoku et al., 1973; Bai and Jin, 1994). Moreover, a group of lipid amides were also putatively detected from the *A. tinctoria* roots samples and represented in cluster B. These amides derivatives were tentatively identified as palmitoleamide (7), linoleamide (8), oleamide (9), and stearamide (10) with protonated molecular ions [M + H]^+^ at *m*/*z* 254, 280, 282, and 284, presenting a loss of a fragment of *m*/*z* 17, corresponding to the loss of an ammoniac molecule (Divito et al., 2012). Other isomeric compounds were also detected by HPLC-HRMS/MS, but in the absence of their fragmentation patterns, putative identities were not proposed. No significant differences were noticed in the metabolites’ production profile of *A. tinctoria* roots associated or not with different AMF strains (Figure 4).

In Experiment 3, shikonin was detected only in negative mode and this result was corroborated by co-injection with shikonin standard solution (Table 4). Due to the important differences in the fragmentation spectra in positive mode of A/Sd, clusters of these compounds were not formed (Figure 5). In addition, the abovementioned shikonin derivatives isomers (1 and 6) were also observed as deprotonated molecular ion [M − H]^–^ at *m*/*z* 301 with fragmentation signals at *m*/*z* 286 and 232 corresponding to the loss of a CH_3_ and C_5_H_9_ groups, for 1, and at *m*/*z* 269 with fragment loss at *m*/*z* 251 and 241, corresponding to the loss of a H_2_O and CO molecules, for 6. The deprotonated molecular ion [M − H]^–^ of shikonin (11) was observed at *m*/*z* 287 with fragmentation signals at *m*/*z* 218 and 190, corresponding to the loss of C_5_H_9_ and the subsequent loss of a CO group (Liao et al., 2015). Moreover, an additional shikonin derivative was observed with a deprotonated molecular ion [M − H]^–^ at *m*/*z* 329, putatively identified as acetylshikonin (13), with fragmentation loss at *m*/*z* 269, 251, and 241 corresponding to a McLafferty rearrangement (i.e., neutral loss of acetic acid), and the subsequent loss of a H_2_O and CO molecules (Liao et al., 2015). Three others unidentified compounds (12, 14, and 15) were detected under negative ionization mode with deprotonated molecular ion [M-H]^–^ at *m*/*z* 797, 1111, and 1147. However, no clusters were observed between compounds 1, 6, 11, and 13 (shikonin derivatives) and for 12, 14, and 15 (unknown compounds) because they show a low degree of similarities in their fragmentation spectra (in positive ionization mode).

Slight differences in metabolites production were observed between Experiments 2 and 3, when samples were analyzed in the same ionization mode. Nevertheless, the most important difference between the two experiments was the detection of shikonin only in Experiment 3. Analyses performed in negative mode for root samples of Experiment 2 did not show the presence of shikonin. Similarly, in Experiment 3, no significant differences were observed in the metabolites’ production profile of *A. tinctoria* roots associated or not with different AMF strains, thus producing similar chromatographic profiles.

## 4. Discussion

In the present study, two native AMF strains isolated from *A. tinctoria* roots and two strains from GINCO were tested and compared on the production of A/Sd and on the expression of genes involved in A/Sd biosynthesis in *A. tinctoria* plants grown either in a semi-hydroponic or in a pot cultivation system. In Experiment 1, conducted only with the GINCO strain *R. irregularis* MUCL 41833, no effect was observed on the production of A/Sd, while a significantly higher relative expression of LePGT2 was observed after 37 days compared to 9 days of growth in the S-H cultivation system. Furthermore, the relative expression of *LePGT1* and *LePGT2* genes were significantly higher in the plants colonized with this AMF compared to the non-mycorrhized ones. In Experiment 2, four AMF strains (two from GINCO: *R. irregularis* MUCL 41833 and *R. aggregatus* MUCL 49408 and two isolated from *A. tinctoria*: *R. irregularis* and *S. viscosum*) were associated to *A. tinctoria* in the S-H cultivation system. The native strain *R. irregularis* significantly increased the production of A/Sd in the roots of *A. tinctoria*, although this was not accompanied by an increased expression of genes involved in the A/Sd biosynthesis pathway. In Experiment 3, the two native strains (*R. irregularis* and *S. viscosum*) and the GINCO one (*R. irregularis* MUCL 41833) were associated to *A. tinctoria* in pots. The native strain *R. irregularis* was confirmed to significantly enhance the content of shikonin and total A/Sd in *A. tinctoria* roots. However, no significant increase in relative genes expression was observed.

### 4.1. The S-H and pot cultivation systems are adequate for growth and AMF colonization of *A. tinctoria*

Whatever the growth system (S-H or pot), an increase in biomass of *A. tinctoria* was observed in the presence as well as absence of AMF. No significant difference was observed in SFW between the plants in the M and NM treatments after 9 days in the S-H cultivation system (Experiments 1 and 2) or after 85 days in the pots cultivation system (Experiment 3). However, a significantly lower RFW and TFW was reported for the plants in the M treatment compared to those in the NM treatment after 37 days in the S-H cultivation system (Experiment 1), unlike the results obtained with *A. officinalis* colonized by *R. irregularis* MUCL 41833 in the same S-H cultivation system (Cartabia et al., 2021). These results are not surprising as it has often been reported that plant growth response to AMF inoculation can vary among AMF species and that the direction (e.g., increase or decrease in plant biomass) and magnitude of the response strongly depends on the combination of plant and AMF taxa (Klironomos, 2003).

Root colonization was observed in each AMF-inoculated plant. However, colonization measured at transfer and after 9 and 37 days in the S-H cultivation system (Experiment 1), decreased steadily, probably because of damages caused to the extraradical mycelium at transfer to the S-H cultivation system with no full recovery and development within the roots throughout the experiment (Cartabia et al., 2021). It is not excluded that this is also a reason for the lower plant biomass reported at the end of Experiment 1 compared to the control, due to an imbalance between the carbon resources transported from plant to the fungus in exchange for nutrients transported from fungus to plant. Interestingly, in both Experiments 2 and 3, the total root colonization was almost similar among the AMF strains, while the percentages of arbuscules were higher in presence of the native *R. irregularis* and *S. viscosum* strains, suggesting that they were better adapted to *A. tinctoria* than the GINCO strains. This has often been reported by comparing commercial strains with local strains; the latter being more prone to rapid and effective colonization (Chenchouni et al., 2020; Wu et al., 2021). Unfortunately, this was not translated into higher plant biomass, probably because of the same justification explained above.

### 4.2. AMF isolated from wild *A. tinctoria* impact the production of A/Sd

The production of A/Sd was not impacted by the GINCO strain *R. irregularis* MUCL 41833 (Experiment 1), whereas a significant increase in production was observed with the native *R. irregularis* strain (Experiments 2 and 3) and an intermediate production between this strain and the GINCO strains with the other native strain *S. viscosum* (Experiment 2). Similarly, no significant effect was observed with the GINCO strains *R. irregularis* MUCL 41833 and *R. aggregatus* MUCL 49408 (Experiments 2 and 3). It cannot be ruled out that these results are related to the fungal genotypes, with the native ones being more prone to stimulate the production of A/Sd than the GINCO ones. The difference between the two native strains further suggests some degree of functional specialization. This is supported by recent studies revealing that various species/strains of AMF can induce different changes in the production of metabolites in the same plant species (Kapoor et al., 2002; Larose et al., 2002; Copetta et al., 2006; Khaosaad et al., 2006; Toussaint et al., 2007; Zubek et al., 2010). For example, Toussaint et al. (2007) showed that *Funneliformis caledonium*^2^ increased rosmarinic acid and caffeic acid production in *O. basilicum*, whereas *Funneliformis mosseae* only increased caffeic acid production. In another study, Jurkiewicz et al. (2010) showed that total phenolic acids accumulation was significantly higher in the roots of *Arnica montana* L. associated with AMF collected from particular plant’s natural stands (in Kurpie or in Karkonosze), while intermediate increase was observed with AMF isolated from other regions (i.e., *R. intraradices* UNIJAG PL24-1 and *R. intraradices* BEG 140 or a mixture composed of the above and of *Funneliformis geosporum* UNIJAG PL 12-2, *Septoglomus constrictum* 265-5 Walker and *F. mosseae* BEG 12). Finally, Frew (2020) reported that the inoculation with a mixture of four commercial species of AMF (*Claroideoglomus etunicatum*, *Funneliformis coronatum*, *F. mosseae*, and *R. irregularis*) had a greater effect on phenolic concentrations in *Hordeum vulgare* L. cv. “Hindmarsh” as compared to a single commercial inoculant (*R. irregularis*). Interestingly, the author also demonstrated that the effects of the commercial mixture was not different from the results obtained with a native multi-species AMF inoculant extracted from field soil. This suggested that a commercial AMF mixture provided little to no additional benefits (Frew, 2020). Conversely, several specialized compounds produced by plants may be synthesized as a chemical defense against the presence of AMF in the roots (Copetta et al., 2006). However, this defense mechanism would be likely to occur with AMF strains that have never been associated to the plant. Our native strains isolated from the wild *A. tinctoria* in their natural habitat are more adapted to the plant and are likely to have evolved as good associates. Natives *R. irregularis* and *S. viscosum* could be better adapted to the presence of A/Sd, which are characterized by different biological activities and by various effects on soil microorganisms (e.g., antifungal activities), and thus better interact with their host plants to regulate and enhance the production of these important therapeutic metabolites (Yan et al., 2019). As reported above, a significant higher AC% was observed with M^Rhiz^ and M^Sept^ treatments in Experiments 2 and 3, respectively. An increase in nutrients uptake via the arbuscules can lead to an enhanced production of precursor compounds, such as NADPH, ATP, acetyl-CoA (mevalonic acid pathway), and pyruvate glyceraldehyde and phosphate (methylerythritol-4-phosphate pathway) that are required for the biosynthesis of various SMs (e.g., terpenoids, phenolic, and alkaloids) (Kapoor et al., 2017). In our study, a higher AC% could have led to an increase in the precursor compounds essential to produce A/Sd. However, this was not confirmed in Experiment 3, where the higher AC% in M^Sept^ treatment was not accompanied by a higher production of A/Sd. If we parallel this to plant biomass, it is well known that the response of plants to inoculation with an AMF may vary depending on the AMF species as previously reported (Klironomos, 2003). It is not excluded that similar effect is observed on metabolites production, although this needs to be demonstrated.

### 4.3. AMF modulate the expression of genes involved in the A/Sd biosynthetic pathway in *A. tinctoria* roots

At the end of Experiment 1, a significantly higher relative expression of *LePGT1* and *LePGT2* genes was observed in *A. tinctoria* associated with *R. irregularis* MUCL 41833 compared to the NM treatment. In addition, a significantly higher relative expression of *LePGT2* was observed in the AMF-colonized plants at day 37 compared to day 9. However, these increases were not accompanied by an enhanced production of A/Sd. Conversely, in Experiment 2, a significant lower relative expression of *LePGT2* was observed in plants associated with the native *R. irregularis* strain, compared with the other AMF and NM treatments, whereas a significantly higher production of A/Sd was observed with this strain. In both Experiments, no differences were reported for *GHQH*. In Experiment 3, no significant differences between the treatments were noted for all the genes tested (data not presented).

Although these results may seem surprising, similar outcomes were reported in the literature. For example, Wang et al. (2014) demonstrated that the application of methyl jasmonate (MeJA) on shikonin-deficient *A. euchroma* cell lines rapidly led to the overexpression of several genes involved in the biosynthesis of A/Sd, including *PGT*, but was not accompanied by a significant higher production of A/Sd. In another study, Ahmad et al. (2022b) showed a significantly higher expression of *LePGT1*, *LePGT2*, and *LeGHQH1* in *Lithospermum officinale* L. roots treated with MeJA, but no higher accumulation of total A/Sd, after eight weeks. Conversely, the same authors (Ahmad et al., 2022a) demonstrated that the bacterium *Chitinophaga* sp. strain R-73072 significantly upregulated *LePGT1* and *LePGT2*, and a cytochrome P450-LeCYP76B101 genes, resulting in a significant enhanced production of total A/Sd in *L. erythrorhizon* roots, after 2-3 weeks.

**FIGURE 6:**
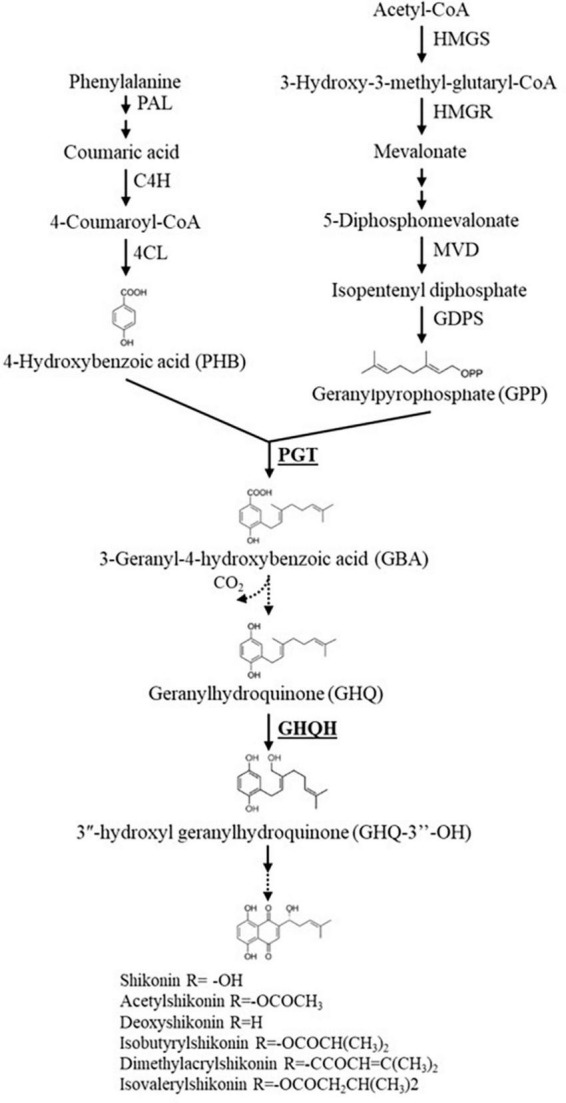
An abridged scheme of the shikonin derivatives biosynthesis pathway. The genes that were investigated in this study are reported in bold font. Single arrows represent one step reaction, while double arrows represent multiple step reactions. Dashed arrows signify undefined steps or the enzymes have not been verified yet. HMGS, 3-hydroxy-3-methylglutaryl-CoA synthase; HMGR, 3-hydroxy-3-methylglutaryl-CoA reductase; MVD, mevalonate diphosphate decarboxylase; GDPS, geranyl diphosphate synthase; PAL, phenylalanine ammonia-lyase; C4H, cinnamic acid 4-hydroxylase; 4CL, 4-coumaroyl-CoA ligase; PGT, *p*-hydroxybenzoate geranyltransferase; GHQH, geranylhydroquinone 3″-hydroxylase.

In our study, a few hypotheses can be advanced to explain why upregulation of *PGT*s was not followed by higher production of A/Sd and, conversely, why downregulation was followed by higher production of A/Sd. Firstly, it is not excluded that the duration of Experiment 1 (37 days) was too short to result in a significant increased production of A/Sd, or that the timing of plant harvesting in our experiments was not optimal (i.e., gene expression behavioral oscillations due to the plant circadian cycle) (Merrow et al., 2005). The *PGT*s regulate the first biosynthetic steps forming the basic carbon skeleton that leads to A/Sd, and therefore, it is possible that the resulting modulation of A/Sd production took longer after the upregulation of the relative expression of target *PGT* genes, as supported by Ahmad et al. (2022b), or that the genes oscillations were not timely targeted during our harvest times, as suggested by Wang et al. (2014) (Figure 6). Secondly, in a study by Andrade et al. (2013), a mismatch between alkaloid levels, another important SM group, and gene expression was reported in different *Catharanthus roseus* (L.) G. Don tissues. In this study, the AMF *C. etunicatum* had a greater influence (i.e., alkaloids production) in roots than in shoots, and a higher gene expression was reported in the older leaves of M plants as well as in the youngest leaves of NM plants (leaves were harvested at the same time) (Andrade et al., 2013). These results suggest a very precise phenological and spatial regulation process during alkaloid biosynthesis (Mahroug et al., 2007). Moreover, the reported influence of AMF on idioblast and laticifer density in *C. roseus* plants might have enhanced the expression of enzymes specifically located in these cells (St-Pierre et al., 1999). An increase in glandular trichome density upon mycorrhization was also linked with an enhanced concentration of another group of SMs, the terpenoids (Zhao et al., 2022). This is an interesting aspect that needs to be further verified. In fact, A/Sd compounds are sequestered as granules in the phospholipid layer and are accumulated in the apoplastic spaces, and they can be found in the cork layer of mature roots (Brigham et al., 1999; Singh et al., 2010; Tatsumi et al., 2016). A difference in A/Sd accumulation might have occurred (i.e., higher content in the primary roots as compared to the secondary roots) upon AMF colonization and might have not been fully displayed across *A. tinctoria* root samples used for our RT-qPCR analysis (i.e., no homogenous samples analyzed with mostly secondary roots utilized). Thirdly, the regulation and accumulation of SMs in plants is usually controlled by a complex network characterized by transcription factors (TFs), which promote or inhibit the expression of multiple genes involved in one or more biosynthetic pathways (Yang et al., 2012; Wu et al., 2021). Transcription factors can act alone or in combination with other TFs to modulate the expression of target genes, and also one TF can regulate the expression of multiple genes participating in one or more biosynthetic pathways (Pinson et al., 2009; Goossens et al., 2017; Hassani et al., 2020). In our study, different TFs might have regulated the target genes (*PGT*s and *GHQH*), yet the full landscape of the A/Sd biosynthesis pathway is not entirely understood, with co-expressed genes still to be identified (Suttiyut et al., 2022). Finally, even after a gene has been transcribed, its expression could still be regulated at various stages. Post transcriptional modifications changes might occur to a newly transcribed primary RNA transcript after transcription has occurred and prior to its translation into a protein.

### 4.4. Chemical profile of *A. tinctoria* does not markedly differ between semi-hydroponic and pot cultivation systems and in presence of different AMF

A similar chemical composition was reported in *A. tinctoria* roots associated with the different AMF and non-colonized control plants in Experiments 2 (S-H cultivation system) and 3 (pot cultivation system) (Figures 4, 5) except for the detection of shikonin in Experiment 3 (Tables 3, 4).

The major compound detected in both experiments was putatively identified as anhydroalkannin (6). This compound is also a main product of shikonin biotransformation by several human intestinal bacteria in aerobic conditions and it was found to be less cytotoxic against a series of human tumor cell lines *in vitro*, in comparison with shikonin (Meselhy et al., 1994; Min et al., 2000). Methyl A/Sd isomers, as detected in our samples, were also identified from *Lithospermum*, *Alkanna*, and *Onosma* species. 1′-methyl shikonin was isolated and characterized from roots of *L. erythrorhizon*, exerting moderate antioxidant activity (Han et al., 2008). To the best of our knowledge, 5- or 8- O-methoxyshikonin derivatives have not yet been identified from natural sources. To better clarify the chemical composition of our samples, putative methylshikonin derivatives (1 and 4) must be purified and the methoxyl position elucidated in the structure of these isomers. In addition, in both experiments, four main lipid amides were tentatively identified as palmitoleamide (7), linoleamide (8), oleamide (9), and stearamide (10). Fatty acids amides are a group of nitrogen-containing, lipid-soluble fatty acid derivatives, which act against a variety of diseases such as cancer, bacterial infections, parasitic infection, inflammations, diabetes, and obesity (Kim et al., 2010; Tanvir et al., 2018). Further, it has been reported that fatty acid amides as oleamide from plant root exudates can participate in strong plant-microbe interactions, stimulating nitrogen metabolism in rhizospheric bacteria (Sun et al., 2016).

The data of chemical composition obtained from *A. tinctoria* growing in our cultivation systems cannot be accurately compared with data from literature (i.e., from nature, commercial samples, and/or from cell plant suspension). Indeed, the proportion and quantity of A/Sd varies depending on the level of stress and microorganisms present in the rhizosphere (Brigham et al., 1999). The influence of the cultivation systems on the production of these methoxy and anhydro A/Sd must be deeply studied to better understand their significance. Secondary metabolites’ production and compositional changes have a strong correlation and association with the environment, and thus synthesized only under specific growth conditions (Peñuelas and Llusià, 1997). Variations in an environmental factor, such as light, temperature, soil water, soil fertility, and salinity, may alter the plant metabolites content (Yang et al., 2018). Indeed, many chemical and physical factors have been found to inhibit shikonin production, such as NH_4_^+^, 2,4-dichlorophenoxyacetic acid (a synthetic auxin), low pH, temperature higher than 28°C, and light, especially blue light (Tatsumi et al., 2016). Skoneczny et al. (2017), using a metabolomic approach profiling hydroxynaphthoquinones (HNQs) and pyrrolizidine alkaloids (PAs), demonstrated the influence of high temperatures and water withholding on the accumulation of A/Sd in *Echium plantagineum* L. Abundance of HNQs, especially deoxyshikonin, shikonin, and dimethylacrylshikonin, rapidly increased in roots exposed to elevated temperatures. Water withholding initially increased NQ abundance, but prolonged drought resulted in reduced total PAs and HNQs (Skoneczny et al., 2017). In our study, *A. tinctoria* growing in the S-H cultivation system were kept in perlite, while a mixture of peatmoss, perlite, and quartz was used in the pots experiment. Perlite is a growing medium frequently used in hydroponic cultivation system since it has a high-water retention and provide the plants’ roots with strong anchor points for stability and strength. Moreover, in this system, plants received minerals by the circulating nutrient solution flowing directly through the plants’ container, while in the pots it was added at constant intervals and left to be completely absorbed by the plants. Therefore, it is possible that the temperature and the water/nutrient solution retention was higher in the conventional pots than in the plant containers of the S-H cultivation system. Moreover, shikonin and A/Sd might have better accumulated in the pots, while leached out in the S-H system. Finally, plant developmental stage qualitatively and quantitatively influences primary and secondary metabolism. A recent study by Csorba et al. (2022) reported this aspect as the most important driver influencing *A. tinctoria* metabolites content, revealing a peak content of A/Sd at the fruiting stage. In our study, plants were harvested during the vegetative growth (around five months old), and thus, might have played a role in the chemical profile reported.

## 5. Conclusion

For the first time, to the best of our knowledge, AMF isolated from wild-growing *A. tinctoria* were identified and applied under the cultivation systems described in this study. Native *R. irregularis* significantly increased A/Sd production in *A. tinctoria* roots, whatever the system used (S-H or pots), thus opening new perspectives toward the application of AMF in the production of these valuable therapeutic compounds in medicinal plants. A better adaptation of this indigenous strain toward its host was demonstrated, with higher arbuscules formation and production of A/Sd reported in *A. tinctoria* roots. This result suggests that the selection of the most effective AMF species (native or not; single or combinations of different AMF strains) remains a key point in studying the modulation/increase of SMs. However, the mechanisms behind AM symbiosis and their impact on the A/Sd biosynthetic pathways still need to be further clarified. Additionally, the conditions characterizing the conventional pots system seemed to be the optimal one in term of shikonin detection. Therefore, the application of the best growing conditions should be further investigated as well. Since shikonin was reported only in the pot system, the recovery of SMs can yet be conducted only in a destructive way (i.e., using the roots of *A. tinctoria*). For this reason, further studies applying S-H cultivation systems/innovative systems are required, especially for testing non-destructive ways of trapping the metabolites exudates by the roots in the circulating nutrient solution.

## Data availability statement

The datasets presented in this study can be found in online repositories. The names of the repository/repositories and accession number(s) can be found in the article/Supplementary material.

## Author contributions

YZ: isolation and identification of AMF from *A. tinctoria* plants, experimental set up, data collection, analysis and interpretation, drafting the work, commentaries, corrections, final approval, and agreement with all aspects of the work. AC: *A. tinctoria in vitro* and *ex vitro* production, experimental setup, data collection, development of protocols for the analyses and interpretation, drafting the work, commentaries, corrections, final approval, and agreement with all aspects of the work. SO: data analysis and interpretation, draft corrections, final approval, and agreement with all aspects of the work. MG-R: contribution on strain identification and assembly of AMF phylogenetic tree. M-FH: data analysis and interpretation, final approval, and agreement with all aspects of the work. JQ-L and SD: substantial contributions to the conception and design of the experiments, interpretation of the data, draft corrections, final approval, and agreement with all aspects of the work. IL: contribution to the development of the experiment, data analysis and interpretation, draft correction, and final approval and agreement with all aspects of the work. All authors contributed to the article and approved the submitted version.

## Footnotes

1
http://www.mycorrhiza.be/ginco-bel/

2 It is to be noticed that the species names of AMF follow the nomenclature of today not the one at the time of publication.

## References

[B1] Ahmad M. Leroy T. Krigas N. Temsch E. M. Weiss-Schneeweiss H. Lexer C. (2021). Spatial and ecological drivers of genetic structure in Greek populations of *Alkanna tinctoria* (*Boraginaceae*), a polyploid medicinal herb. *Front. Plant Sci.* 12:706574. 10.3389/fpls.2021.706574 34335669 PMC8317432

[B2] Ahmad M. Varela A. A. Koletti A. E. Rodić N. Reichelt M. Rödel P. (2022b). Dynamics of alkannin/shikonin biosynthesis in response to jasmonate and salicylic acid in *Lithospermum officinale*. *Sci Rep.* 12:17093. 10.1038/s41598-022-21322-0 36224205 PMC9554848

[B3] Ahmad M. Varela A. A. Koletti A. E. Assimopoulou A. N. Declerck S. Schneider C. (2022a). Transcriptional dynamics of *Chitinophaga* sp. strain R-73072-mediated alkannin/shikonin biosynthesis in *Lithospermum officinale*. *Front. Microbiol.* 13:978021. 10.3389/fmicb.2022.978021 36071973 PMC9441710

[B4] Andrade S. A. L. Malik S. Sawaya A. C. H. F. Bottcher A. Mazzafera P. (2013). Association with arbuscular mycorrhizal fungi influences alkaloid synthesis and accumulation in *Catharanthus roseus* and *Nicotiana tabacum* plants. *Acta Physiol Plant*. 35 867–880. 10.1007/s11738-012-1130-8

[B5] Avio L. Turrini A. Giovannetti M. Sbrana C. (2018). Designing the ideotype mycorrhizal symbionts for the production of healthy food. *Front. Plant Sci*. 9:1089. 10.3389/fpls.2018.01089 30154803 PMC6102486

[B6] Bai G. Jin X. J. (1994). Chemical constituents of *Lithospermum erythrorhizon*. *Chem. Res. Chin. Univ.* 10 263–265.

[B7] Bossard E. Tsafantakis N. Aligiannis N. Fokialakis N. (2022). A development strategy of tailor-made natural deep eutectic solvents for the enhanced extraction of hydroxynaphthoquinones from *Alkanna tinctoria* roots. *Planta Med.* 88 826–837. 10.1055/a-1738-5648 35021247

[B8] Brigham L. A. Michaels P. J. Flores H. E. (1999). Cell-specific production and antimicrobial activity of naphthoquinones in roots of *Lithospermum erythrorhizon*. *Plant Physiol.* 119 417–428. 10.1104/pp.119.2.417 9952436 PMC32117

[B9] Cartabia A. Sarropoulou V. Grigoriadou K. Maloupa E. Declerck S. (2022). *In vitro* propagation of *Alkanna tinctoria* Tausch.: A medicinal plant of the *Boraginaceae* family with high pharmaceutical value. *Ind. Crops Prod.* 182:114860. 10.1016/j.indcrop.2022.114860

[B10] Cartabia A. Tsiokanos E. Tsafantakis N. Lalaymia I. Termentzi A. Miguel M. (2021). The arbuscular mycorrhizal fungus *Rhizophagus irregularis* MUCL 41833 modulates metabolites production of *Anchusa officinalis* L. under semi-hydroponic cultivation. *Front. Plant Sci.* 12:724352. 10.3389/fpls.2021.724352 34539717 PMC8443025

[B11] Chaudhary V. Kapoor R. Bhatnagar A. K. (2008). Effectiveness of two arbuscular mycorrhizal fungi on concentrations of essential oil and artemisinin in three accessions of *Artemisia annua* L. *Appl. Soil Ecol.* 40 174–181. 10.1016/j.apsoil.2008.04.003

[B12] Chen M. Arato M. Borghi L. Nouri E. Reinhardt D. (2018). Beneficial services of arbuscular mycorrhizal fungi – From ecology to application. *Front. Plant Sci.* 9:1270. 10.3389/fpls.2018.01270 30233616 PMC6132195

[B13] Chenchouni H. Mekahlia M. N. Beddiar A. (2020). Effect of inoculation with native and commercial arbuscular mycorrhizal fungi on growth and mycorrhizal colonization of olive (*Olea europaea* L.). *Sci. Hortic. Vol.* 261:108969. 10.1016/j.scienta.2019.108969

[B14] Copetta A. Lingua G. Berta G. (2006). Effects of three AM fungi on growth, distribution of glandular hairs, and essential oil production in *Ocimum basilicum* L. var. Genovese. *Mycorrhiza* 16 485–494. 10.1007/s00572-006-0065-6 16896796

[B15] Csorba C. Rodić N. Zhao Y. Antonielli L. Brader G. Vlachou A. (2022). Metabolite production in *Alkanna tinctoria* links plant development with the recruitment of individual members of microbiome thriving at the root-soil interface. *mSystems* 7 e451–e422. 10.1128/msystems.00451-22 36069453 PMC9601132

[B16] Divito E. B. Davic A. P. Johnson M. E. Cascio M. (2012). Electrospray ionization and collision induced dissociation mass spectrometry of primary fatty acid amides. *Anal. Chem.* 84 2388–2394. 10.1021/ac203158u 22283789

[B17] Frew A. (2020). Contrasting effects of commercial and native arbuscular mycorrhizal fungal inoculants on plant biomass allocation, nutrients and phenolics. *Plants People Planet* 3 536–540. 10.1002/ppp3.10128

[B18] Garcés-Ruiz M. Calonne-Salmon M. Plouznikoff K. Misson C. Navarrete-Mier M. Cranenbrouck S. (2017). Dynamics of short-term phosphorus uptake by intact mycorrhizal and non-mycorrhizal maize plants grown in a circulatory semi-hydroponic cultivation system. *Front. Plant Sci.* 8:1471. 10.3389/fpls.2017.01471 28890723 PMC5574913

[B19] Gerardi C. Mita G. Grillo E. Giovinazzo G. Miceli A. De Leo P. (1998). “*Alkanna tinctoria* T. (Alkanets): *In vitro* culture and the production of alkannin and other secondary metabolites,” in *Medicinal and aromatic plants X, biotechnology in agriculture and forestry*, ed. Bajaj Y. P. S. (Berlin: Springer Berlin Heidelberg), 10.1007/978-3-642-58833-4_2

[B20] Gontier E. Clément A. Tran T. L. M. Gravot A. Lièvre K. Guckert A. (2002). Hydroponic combined with natural or forced root permeabilization: A promising technique for plant secondary metabolite production. *Plant Sci.* 163 723–732. 10.1016/S0168-9452(02)00171-1

[B21] Goossens J. Mertens J. Goossens A. (2017). Role and functioning of bHLH transcription factors in jasmonate signalling. *J. Exp. Bot.* 68 1333–1347. 10.1093/jxb/erw440 27927998

[B22] Gupta K. Garg S. Singh J. Kumar M. (2014). Enhanced production of napthoquinone metabolite (shikonin) from cell suspension culture of *Arnebia* sp. and its up-scaling through bioreactor. *3 Biotech* 4 263–273. 10.1007/s13205-013-0149-x 28324426 PMC4026457

[B23] Han J. Weng X. C. Bi K. (2008). Antioxidants from a Chinese medicinal herb – *Lithospermum erythrorhizon*. *Food Chemistry* 106 2–10. 10.1016/j.foodchem.2007.01.031

[B24] Hassani D. Fu X. Shen Q. Khalid M. Rose J. K. C. Tang K. (2020). Parallel transcriptional regulation of artemisinin and flavonoid biosynthesis. *Trends Plant Sci.* 25 466–476. 10.1016/j.tplants.2020.01.001 32304658

[B25] Hubert P. Nguyen-Huu J. J. Boulanger B. Chapuzet E. Chiap P. Cohen N. (2003). Validation des procédures analytiques quantitatives, Harmonisation des démarches. *S.T.P. Pharma Pratiq.* 13 101–138.

[B26] IJdo M. Cranenbrouck S. Declerck S. (2011). Methods for large-scale production of AM fungi: Past, present, and future. *Mycorrhiza* 21 1–16. 10.1007/s00572-010-0337-z 20803040

[B27] Jurkiewicz A. Ryszka P. Anielska T. Waligórski P. Białońska D. Góralska K. (2010). Optimization of culture conditions of *Arnica montana* L.: Effects of mycorrhizal fungi and competing plants. *Mycorrhiza* 20 293–306. 10.1007/s00572-009-0280-z 19838743

[B28] Kapoor R. Anand G. Gupta P. Mandal S. (2017). Insight into the mechanisms of enhanced production of valuable terpenoids by arbuscular mycorrhiza. *Phytochem Rev.* 16 677–692. 10.1007/s11101-016-9486-9

[B29] Kapoor R. Giri B. Mukerji K. G. (2002). *Glomus macrocarpum*: A potential bioinoculant to improve essential oil quality and concentration in Dill (*Anethum graveolens* L.) and Carum (*Trachyspermum ammi* (Linn.) Sprague). *World J. Microbiol. Biotechnol.* 18 459–463. 10.1023/A:1015522100497

[B30] Kaur S. Suseela V. (2020). Unraveling arbuscular mycorrhiza-induced changes in plant primary and secondary metabolome. *Metabolites* 10:335. 10.3390/metabo10080335 32824704 PMC7464697

[B31] Khaosaad T. Vierheilig H. Nell M. Zitterl-Eglseer K. Novak J. (2006). Arbuscular mycorrhiza alter the concentration of essential oils in oregano (*Origanum sp.*, Lamiaceae). *Mycorrhiza* 16 443–446. 10.1007/s00572-006-0062-9 16909287

[B32] Kim K. H. Choi S. U. Son M. W. Lee K. R. (2010). Two new phenolic amides from the seeds of pharbitis nil. *Chem. Pharm Bull*. 58 1532–1535. 10.1248/cpb.58.1532 21048350

[B33] Klironomos J. (2003). Variation in plant response to native and exotic arbuscular mycorrhizal fungi. *Ecology*. 84 2292–2301. 10.1890/02-0413

[B34] Kyogoku K. Terayama H. Tachi Y. Suzuki T. Komatsu M. (1973). Studies on the constituents of “shikon”. I. Structure of three new shikonin derivatives and isolation of anhydroalkanin. *Shoyakugaku Zasshi*. 27 31–36.

[B35] Larose G. Chênevert R. Moutoglis P. Gagné S. Piché Y. Vierheilig H. (2002). Flavonoid levels in roots of *Medicago sativa* are modulated by the developmental stage of the symbiosis and the root colonizing arbuscular mycorrhizal fungus. *J. Plant Physiol.* 159 1329–1339. 10.1078/0176-1617-00896

[B36] Liao M. Li A. Chen C. Ouyang H. Zhang Y. Xu Y. (2015). Systematic identification of shikonins and shikonofurans in medicinal Zicao species using ultra-high performance liquid chromatography quadrupole time of flight tandem mass spectrometry combined with a data mining strategy. *J. Chromatogr. A.* 1425 158–172. 10.1016/j.chroma.2015.11.028 26610615

[B37] Lu F. C. Lee C. Y. Wang C. L. (2015). The influence of arbuscular mycorrhizal fungi inoculation on yam (*Dioscorea* spp.) tuber weights and secondary metabolite content. *PeerJ* 3 e1266. 10.7717/peerj.1266 26421239 PMC4586806

[B38] Mahroug S. Burlat V. St-Pierre B. (2007). Cellular and sub-cellular organisation of the monoterpenoid indole alkaloid pathway in *Catharanthus roseus*. *Phytochem. Rev.* 6 363–381. 10.1007/s11101-006-9017-1

[B39] Malik S. Bhushan S. Sharma M. Ahuja P. S. (2016). Biotechnological approaches to the production of shikonins: A critical review with recent updates. *Crit. Rev. Biotechnol.* 36 327–340. 10.3109/07388551.2014.961003 25319455

[B40] McGonigle T. P. Miller M. H. Evans D. G. Fairchild G. L. Swan J. A. (1990). A new method which gives an objective measure of colonization of roots by vesicular-arbuscular mycorrhizal fungi. *New Phytol.* 115 495–501. 10.1111/j.1469-8137.1990.tb00476.x 33874272

[B41] Merrow M. Spoelstra K. Roenneberg T. (2005). The circadian cycle: Daily rhythms from behaviour to genes: First in the cycles review series. *EMBO Rep*. 6 930–935. 10.1038/sj.embor.7400541 16222241 PMC1369194

[B42] Meselhy R. M. Shigetoshi K. Koji T. Masao H. Tsuneo N. (1994). Biotransformation of shikonin by human intestinal bacteria. *Tetrahedron* 50 3081–3098. 10.1016/S0040-4020(01)81108-X

[B43] Min B. S. Hattori M. Kim H. M. Kim Y. H. (2000). Cytotoxicity of shikonin metabolites with biotransformation of human intestinal bacteria. *J. Microbial. Biotechnol.* 10 514–517.

[B44] Pandey D. K. Kaur P. Dey A. (2018). “Arbuscular mycorrhizal fungi: Effects on secondary metabolite production in medicinal plants,” in *Fungi and their role in sustainable development: Current perspectives*, eds Gehlot P. Singh J. (Singapore: Springer Singapore), 10.1007/978-981-13-0393-7_28

[B45] Papageorgiou V. Assimopoulou A. Ballis A. (2008). Alkannins and shikonins: A new class of wound healing agents. *CMC* 15 3248–3267. 10.2174/092986708786848532 19075667

[B46] Papageorgiou V. P. Assimopoulou A. N. Couladouros E. A. Hepworth D. Nicolaou K. C. (1999). The chemistry and biology of alkannin, shikonin, and related naphthazarin natural products. *Angew Chem. Int. Ed. Engl.* 38 270–301. 29711637 10.1002/(SICI)1521-3773(19990201)38:3<270::AID-ANIE270>3.0.CO;2-0

[B47] Peñuelas J. Llusià J. (1997). Effects of carbon dioxide, water supply, and seasonally on terpene content and emission by *Rosmarinus officinalis*. *J. Chem. Ecol.* 23 979–993. 10.1023/B:JOEC.0000006383.29650.d7

[B48] Pfaffl M. W. (2001). A new mathematical model for relative quantification in real-time RT-PCR. *Nucleic Acids Res.* 29 45e–445e. 10.1093/nar/29.9.e45 11328886 PMC55695

[B49] Pinson B. Vaur S. Sagot I. Coulpier F. Lemoine S. Daignan-Fornier B. (2009). Metabolic intermediates selectively stimulate transcription factor interaction and modulate phosphate and purine pathways. *Genes Dev.* 23 1399–1407. 10.1101/gad.521809 19528318 PMC2701576

[B50] Pluskal T. Castillo S. Villar-Briones A. Oresic M. (2010). MZmine 2: Modular framework for processing, visualizing, and analyzing mass spectrometry-based molecular profile data. *BMC Bioinform.* 11:395. 10.1186/1471-2105-11-395 20650010 PMC2918584

[B51] Sgherri C. Cecconami S. Pinzino C. Navari-Izzo F. Izzo R. (2010). Levels of antioxidants and nutraceuticals in basil grown in hydroponics and soil. *Food Chem*. 123 416–422. 10.1016/j.foodchem.2010.04.058

[B52] Shannon P. Markiel A. Ozier O. Baliga N. S. Wang J. T. Ramage D. (2003). Cytoscape: A software environment for integrated models of biomolecular interaction networks. *Genome Res.* 13 2498–2504. 10.1101/gr.1239303 14597658 PMC403769

[B53] Singh R. S. Gara R. K. Bhardwaj P. K. Kaachra A. Malik S. Kumar R. (2010). Expression of 3-hydroxy-3-methylglutaryl-CoA reductase, p-hydroxybenzoate-m-geranyltransferase and genes of phenylpropanoid pathway exhibits positive correlation with shikonins content in arnebia [*Arnebia euchroma* (Royle) Johnston]. *BMC Mol. Biol.* 11:88. 10.1186/1471-2199-11-88 21092138 PMC3002352

[B54] Skoneczny D. Weston P. A. Zhu X. Gurr G. M. Callaway R. M. Barrow R. A. (2017). Metabolic profiling and identification of shikonins in root periderm of two invasive *Echium* spp. weeds in Australia. *Molecules* 22:330. 10.3390/molecules22020330 28230806 PMC6155885

[B55] Smith S. E. Read D. (2008). *Mineral nutrition, toxic element accumulation and water relations of arbuscular mycorrhizal plants.* Amsterdam: Elsevier, 10.1016/B978-012370526-6.50007-6

[B56] Song W. Zhuang Y. Liu T. (2020). Potential role of two cytochrome P450s obtained from *Lithospermum erythrorhizon* in catalyzing the oxidation of geranylhydroquinone during Shikonin biosynthesis. *Phytochemistry* 175:112375. 10.1016/j.phytochem.2020.112375 32305685

[B57] St-Pierre B. Vazquez-Flota F. A. De Luca V. (1999). Multicellular compartmentation of *Catharanthus roseus* alkaloid biosynthesis predicts intercellular translocation of a pathway intermediate. *Plant Cell* 11 887–900. 10.1105/tpc.11.5.887 10330473 PMC144229

[B58] Sun L. Lu Y. Kronzucker H. J. Shi W. (2016). Quantification and enzyme targets of fatty acid amides from duckweed root exudates involved in the stimulation of denitrification. *J. Plant Physiol.* 198 81–88. 10.1016/j.jplph.2016.04.010 27152459

[B59] Suttiyut T. Auber R. P. Ghaste M. Kane C. N. McAdam S. A. M. Wisecaver J. H. (2022). Integrative analysis of the shikonin metabolic network identifies new gene connections and reveals evolutionary insight into shikonin biosynthesis. *Hortic. Res.* 9:uhab087. 10.1093/hr/uhab087 35048120 PMC8969065

[B60] Takanashi K. Nakagawa Y. Aburaya S. Kaminade K. Aoki W. Saida-Munakata Y. (2019). Comparative proteomic analysis of *Lithospermum erythrorhizon* reveals regulation of a variety of metabolic enzymes leading to comprehensive understanding of the shikonin biosynthetic Pathway. *Plant Cell Physiol.* 60 19–28. 10.1093/pcp/pcy183 30169873

[B61] Tanvir R. Javeed A. Rehman Y. (2018). Fatty acids and their amide derivatives from endophytes: New therapeutic possibilities from a hidden source. *FEMS Microbiol. Lett.* 365:fny114. 10.1093/femsle/fny114 29733374

[B62] Tappeiner J. Vasiliou A. Ganzera M. Fessas D. Stuppner H. Papageorgiou V. P. (2014). Quantitative determination of alkannins and shikonins in endemic Mediterranean *Alkanna* species: Quantitative determination of alkannins and shikonins. *Biomed. Chromatogr.* 28 923–933. 10.1002/bmc.3096 24327564

[B63] Tatsumi K. Yano M. Kaminade K. Sugiyama A. Sato M. Toyooka K. (2016). Characterization of shikonin derivative secretion in *Lithospermum erythrorhizon* hairy roots as a model of lipid-soluble metabolite secretion from plants. *Front. Plant Sci.* 7:1066. 10.3389/fpls.2016.01066 27507975 PMC4961010

[B64] Toussaint J.-P. Smith F. A. Smith S. E. (2007). Arbuscular mycorrhizal fungi can induce the production of phytochemicals in sweet basil irrespective of phosphorus nutrition. *Mycorrhiza* 17 291–297. 10.1007/s00572-006-0104-3 17273856

[B65] Tsiokanos E. Cartabia A. Tsafantakis N. Lalaymia I. Termentzi A. Miguel M. (2022). The metabolic profile of *Anchusa officinalis* L. differs according to its associated arbuscular mycorrhizal fungi. *Metabolites* 12:573. 10.3390/metabo12070573 35888697 PMC9319164

[B66] Urbanek H. Bergier K. Saniewski M. Patykowski J. (1996). Effect of jasmonates and exogenous polysaccharides on production of alkannin pigments in suspension cultures of *Alkanna tinctoria*. *Plant Cell Rep*. 15 637–641. 10.1007/BF00232468 24178533

[B67] Valdés B. (2011). *Boraginaceae in Euro+Med plantbase: The information resource for Euro-Mediterranean plant diversity.* Poltava: EuroPlus.

[B68] Walker C. (2005). A simple blue staining technique for arbuscular mycorrhizal and other root-inhabiting fungi. *Inoculum* 56 68–69.

[B69] Wang M. Carver J. J. Phelan V. V. Sanchez L. M. Garg N. Peng Y. (2016). Sharing and community curation of mass spectrometry data with global natural products social molecular networking. *Nat. Biotechnol.* 34 828–837. 10.1038/nbt.3597 27504778 PMC5321674

[B70] Wang S. Guo L. P. Xie T. Yang J. Tang J. F. Li X. (2014). Different secondary metabolic responses to MeJA treatment in shikonin-proficient and shikonin-deficient cell lines from *Arnebia euchroma* (Royle) Johnst. *Plant Cell Tiss. Organ. Cult.* 119 587–598. 10.1007/s11240-014-0558-5

[B71] Wu F. Y. Tang C. Y. Guo Y. M. Bian Z. W. Fu J. Y. Lu G. H. (2017). Transcriptome analysis explores genes related to shikonin biosynthesis in Lithospermeae plants and provides insights into boraginales’ evolutionary history. *Sci. Rep.* 7:4477. 10.1038/s41598-017-04750-1 28667265 PMC5493674

[B72] Wu Y. H. Wang H. Liu M. Li B. Chen X. Ma Y. T. (2021). Effects of native arbuscular mycorrhizae isolated on root biomass and secondary metabolites of *Salvia miltiorrhiza* Bge. *Front. Plant Sci.* 12:617892. 10.3389/fpls.2021.617892 33603763 PMC7884620

[B73] Xu J. Aileni M. Abbagani S. Zhang P. (2010). A reliable and efficient method for total RNA isolation from various members of spurge family (*Euphorbiaceae*). *Phytochem. Anal.* 21 395–398. 10.1002/pca.1205 20135710

[B74] Yamamoto H. Inoue K. Li S. M. Heide L. (2000). Geranylhydroquinone 3”-hydroxylase, a cytochrome P-450 monooxygenase from *Lithospermum* erythrorhizon cell suspension cultures. *Planta* 210 312–317. 10.1007/PL00008139 10664138

[B75] Yaman C. Uranbey S. Ahmed H. A. Ozcan S. Tugay O. Basalma D. (2019). Callus induction and regeneration of *Alkanna orientalis* var. orientalis and *A. sieheana*. *Bangladesh J. Bot.* 48 633–640. 10.3329/bjb.v48i3.47941

[B76] Yan Y. Tan F. Miao H. Wang H. Cao Y. (2019). Effect of shikonin against *Candida albicans* biofilms. *Front Microbiol.* 10:1085. 10.3389/fmicb.2019.01085 31156594 PMC6527961

[B77] Yang C. Q. Fang X. Wu X. M. Mao Y. B. Wang L. J. Chen X. Y. (2012). Transcriptional regulation of plant secondary metabolism. *F. J. Integr. Plant Biol.* 54 703–712. 10.1111/j.1744-7909.2012.01161.x 22947222

[B78] Yang L. Wen K. S. Ruan X. Zhao Y. X. Wei F. Wang Q. (2018). Response of plant secondary metabolites to environmental factors. *Molecules* 23 762. 10.3390/molecules23040762 29584636 PMC6017249

[B79] Yazaki K. (2017). *Lithospermum erythrorhizon* cell cultures: Present and future aspects. *Plant Biotechnol*. 34 131–142. 10.5511/plantbiotechnology.17.0823a 31275019 PMC6565996

[B80] Yazaki K. Kunihisa M. Fujisaki T. Sato F. (2002). Geranyl diphosphate:4-Hydroxybenzoate geranyltransferase from *Lithospermum erythrorhizon*. *J. Biol. Chem.* 277 6240–6246. 10.1074/jbc.M106387200 11744717

[B81] Zeng Y. Guo L.-P. Chen B.-D. Hao Z.-P. Wang J.-Y. Huang L.-Q. (2013). Arbuscular mycorrhizal symbiosis and active ingredients of medicinal plants: Current research status and prospectives. *Mycorrhiza* 23 253–265. 10.1007/s00572-013-0484-0 23417725

[B82] Zhao Y. Cartabia A. Lalaymia I. Declerck S. (2022). Arbuscular mycorrhizal fungi and production of secondary metabolites in medicinal plants. *Mycorrhiza* 32 221–256. 10.1007/s00572-022-01079-0 35556179 PMC9184413

[B83] Zubek S. Stojakowska A. Anielska T. Turnau K. (2010). Arbuscular mycorrhizal fungi alter thymol derivative contents of *Inula ensifolia* L. *Mycorrhiza* 20 497–504. 10.1007/s00572-010-0306-6 20177715

