# Supplementary material for: Arbuscular mycorrhizal fungi impact the production of alkannin/shikonin and their derivatives in Alkanna tinctoria Tausch. grown in semi-hydroponic and pot cultivation systems
Source: Front Microbiol. 2023 Aug 10;14:1216029. doi: 10.3389/fmicb.2023.1216029 (PMC10447974; doi:10.3389/fmicb.2023.1216029)
Supplement: Supplementary file 1 [file Data_Sheet_1.docx]

***Supplementary Material***

**Arbuscular mycorrhizal fungi impact the production of alkannin/shikonin and their derivatives in *Alkanna tinctoria* Tausch. grown in semi-hydroponic and pot cultivation systems**

Yanyan Zhao, Annalisa Cartabia, Mónica Garcés-Ruiz, Marie-France Herent, Joëlle Quetin-Leclercq, Sergio Ortiz, Stéphane Declerck^*^, Ismahen Lalaymia

Stéphane Declerck

[stephan.declerck@uclouvain.be](mailto:stephan.declerck@uclouvain.be)

**Isolation and mono-species culture of AMF**

***Evaluation of the presence of AMF in root/soil samples***

Roots of *Alkanna tinctoria* were sampled from the wild in Greece and stained following the protocol of Vierheilig et al. (1998), and Walker (2005), to evaluate root colonization (**Figure S1A**). In parallel, rhizospheric soils were sampled to collect AMF spores applying a procedure adapted from Cranenbrouck et al. (2005) and Walker (2009) (personal communication) (**Figure S1B**). Spores were subsequently mounted on slide with polyvinyl alcohol-lacto-glycerol (PVLG) solution (Omar et al., 1979) and checked under stereomicroscope (Olympus BH2–RFCA, Japan) to confirm AMF presence.


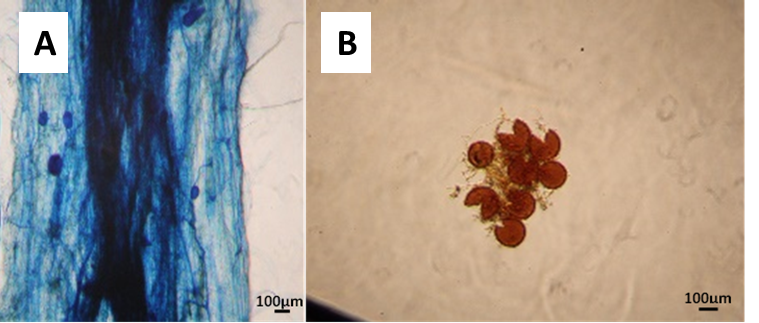


**Figure S1**. (**A**) Stained A. tinctoria root and (**B**) spores, collected from the wild in Greece (Photographs taken with Canon EOS 60D, through the lens of a bright field light stereomicroscope, Olympus BH2-RFCA, Japan).

***Establishment of AMF trap cultures***

Seeds of *Plantago lanceolata* L. (Ecosem, Belgium) and *Medicago truncatula* Gaertn. (SARDI, Australia) were surface sterilized by soaking in 70% ethanol for 1 min and in sodium hypochlorite (8% active chloride) for 5 min, and subsequently rinsed 3 times with deionized water. The seeds were then germinated in a 1.3 L pot (15×11.4 cm) filled with a sterilized (121°C for 15 min) substrate that consist of a mixture of calcinated clay (DCM, Belgium), quartz (0.4-0.8 mm), quartz (1-2 mm), 2:2:1 w/w.

*A. tinctoria* plant rhizospheric soils and roots pieces were used in trap cultures. For roots, thin fragments were placed near the root system of 15-day old seedlings of *M. truncatula* and *P. lanceolata.* Two and 5 seedlings, respectively, were placed in each pot (1.3L, 15×11.4 cm) filled with the same sterilized substrate mixture as above. For rhizospheric soils, samples were placed in individual pots between two layers of the same sterilized substrate as above, and in contact with 15-day old *M. truncatula* and *P. lanceolate* seedlings. In total 24 trap cultures were established (12 for rhizospheric soils and 12 for root samples).

To maintain the trap cultures, the pots were watered to field capacity and supplemented with slow release NPK beads (four beads per pot) (Osmocote PRO 5-6 months, 17-11-10+2MgO+TE, or Osmocote PRO 16-3-11+3 MgO, Everris, France). The pots were maintained in Sunbags (Sigma-Aldrich, Germany) closed with plastic paper clips (Laurel, Germany). They were watered with deionized water every three weeks and kept under greenhouse condition set at 20°C, 50% RH, photoperiod of 16 h day^-1^ and PPF of 96 μmol m^-2^s^-1^ (Figure S2).


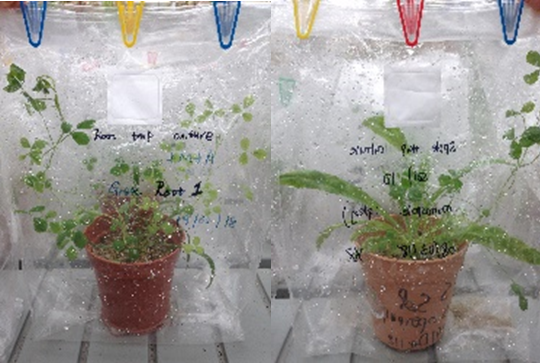


**Figure S2**. Trap cultures of rhizospheric soils with M. truncatula and P. lanceolata plants, under greenhouse conditions (UCLouvain).

***Establishment of AMF mono-species pot cultures***

Roots and substrates from the trap cultures above were used to collect new, actively growing and healthy AMF spores. Roots and substrates were sieved through a sequence of sieves (i.e., 250, 106 and 38 μm, Euromatest Sintco, France) and supernatant cleaned with tap water. Different fractions from the sieves were collected in Petri dishes (50 mm) and observed under stereomicroscope (Olympus, SZ61) to collect healthy-looking spores with tweezer and needles. Single spores or cluster of spores attached by a common mycelium were placed at the intersection between a main root and newly growing secondary root of 15-day old *P. lanceolata* seedlings. Then, the seedlings were placed in 7x7x6 cm pots filled with sterilized substrate mixture as above. Four pots were placed in one closed Sunbag and watered with low-P Hoagland solution (Hoagland and Arnon, 1950) 100x diluted every 20 days. Sunbags were maintained under greenhouse conditions set as above (Figure S3). In total, around 220 single spores AMF pot cultures were established.


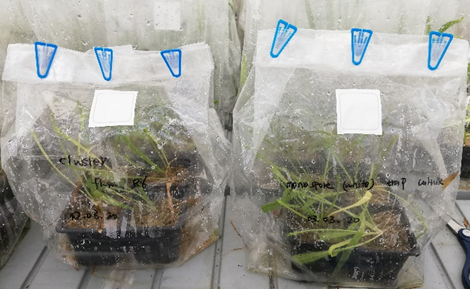


**Figure S3**. Single spores AMF trap cultures with P. lanceolata host plants maintained under greenhouse conditions (UCLouvain).

**Molecular identification of isolated AMF**

In total 44 cultures of AMF were successful. A selection of 9 cultures (with contrasting spores morphologies) were used for molecular identification. Three to 5 healthy spores were collected in each pot by wet sieving and picked with needle and tweezer (Vomm 113 SA). They were used for DNA extraction. They were transferred in 0.2 mL PCR tubes (SARSTEDT, Germany) with sterile needles (AGANI™ NEEDLE, 0.45×23 mm, TERUMO) under laminar flow hood. Five μL of PCR water was added to each sample to crush the spores and obtain the starting 5 μL of template DNA. Two-step PCR applying specific AMF primers amplifying a part of the SSU rRNA gene, the complete ITS region (including the 5.8S rRNA gene) and approx. 800 bp of the LSU rRNA gene, named SSU-ITS-LSU, were used (Krüger et al., 2009). For the first PCR, primer pairs mixtures SSUmAf – LSUmAr were used, and for the nested PCR, primer pairs mixtures SSUmCf – LSUmBr (Table S1) were used. In both amplifications, the reaction mix contained Phusion High Fidelity PCR Master Mix with HF Buffer (Thermo Fisher Scientific, Lithuania) with 0.5 μM concentration of each primer (Sigma, Germany) and 0.2 μg mL^-1^ BSA (Albumin Bovine, AMRESCO, United States). In the first PCR reaction mix, 5 μL of template DNA (as above), 10 μl of Phusion High-Fidelity PCR Master Mix, 1 μL of SSUmAf and LSUmAr, and 3 μL of ultra clean water (Sigma) were used in 20 μL of final reaction. Thermal cycling was performed in an Eppendorf Master-cycler Gradient (Eppendorf Nexus X2, Germany) using the following parameters for the first PCR: initial denaturation of 5 min at 99°C, followed by 40 cycles of denaturation (10 s at 99°C)/ annealing (30 s at 60°C)/ elongation (1 min at 72°C), and a final elongation of 10 min at 72°C. For the nested PCR, 1 μL of the first PCR product was used as template in the same final reaction volume (20 μL) as above, and the same thermal cycling conditions were set up with slight differences: 30 cycles and annealing temperature of 63°C. The PCR products were visualized on 1.0% agarose gel with 1× TAE buffer (Tris/Acetic Acid/EDTA buffer 50x, Carl Roth) and stained with GelRed^®^ (Biotium, USA).

**Table S1:** Forward and reverse primers sequences used to amplify AMF following Krüger et al. (2009).

|  | **Primer** | **Nucleotide sequence (5´-3´)** | **Primer mixtures** |
| --- | --- | --- | --- |
| **First PCR** | SSUmAf1 TGGGTAATCTTTTGAAACTTYA  SSUmAf2 TGGGTAATCTTRTGAAACTTCA | | SSUmAf: mixSSUmAf1-2  (equimolar) |
|  | LSUmAr1 GCTCACACTCAAATCTATCAAA  LSUmAr2 GCTCTAACTCAATTCTATCGAT  LSUmAr3 TGCTCTTACTCAAATCTATCAAA  LSUmAr4 GCTCTTACTCAAACCTATCGA | | LSUmAr: mixLSUmAr1-4 (equimolar) |
| **Nested**  **PCR** | SSUmCf1 TCGCTCTTCAACGAGGAATC  SSUmCf2 TATTGTTCTTCAACGAGGAATC  SSUmCf3 TATTGCTCTTNAACGAGGAATC | | SSUmCf: mixSSUmCf1-3 (equimolar) |
|  | LSUmBr1 DAACACTCGCATATATGTTAGA  LSUmBr2 AACACTCGCACACATGTTAGA  LSUmBr3 AACACTCGCATACATGTTAGA  LSUmBr4 AAACACTCGCACATATGTTAGA  LSUmBr5 AACACTCGCATATATGCTAGA | | LSUmBr: mixLSUmBr1-5 (equimolar) |

Cloning protocol was followed as described by Krüger et al. (2009). Briefly, the 1.5 kb fragments from the nested PCR products was cloned with the Zero Blunt TOPO PCR Cloning Kit (Invitrogen, United States) following the manufacturer’s protocol. Five colonies of each sample were analysed for correct length of plasmid inserts by colony-PCR using 1x GoTaq DNA Polymerase (Promega, United States) and M13F-M13R primers. Correct clone PCR products were sequenced using M13F-M13R primers at Macrogen Inc. (Korea).

Sequences were assembled and edited with software Sequencher 5.4.6 version (Gene Codes Corporation, USA). Homologous sequences were searched by blastn at the National Center for Biotechnology Information (NCBI). An AMF freely available reference alignment from Krüger et al. (2012), was also applied to compare and establish the phylogenetic tree. Assembled sequences, homologous environmental sequences from the NCBI platform and reference alignment were aligned and performed with MAFFT online (Katoh and Standley, 2013) and then manual adjusted at the Phylogenetic Data Editor (PhyDE).

A maximum-likelihood phylogenetic tree was assembled with references using RAxML-HPC2 (Stamatakis et al., 2008) on XSEDE ver. 8.2.9 on the CIPRES Science Gateway6 with 1000 bootstrap and the GTRGAMMA model (Krüger et al., 2012). Taxonomic annotations followed the classification of Schüßler and Walker (2010). The AMF species selected in the paper were closest to *Rhizophagus irregularis* and *Septoglomus viscosum* (Figure S4).


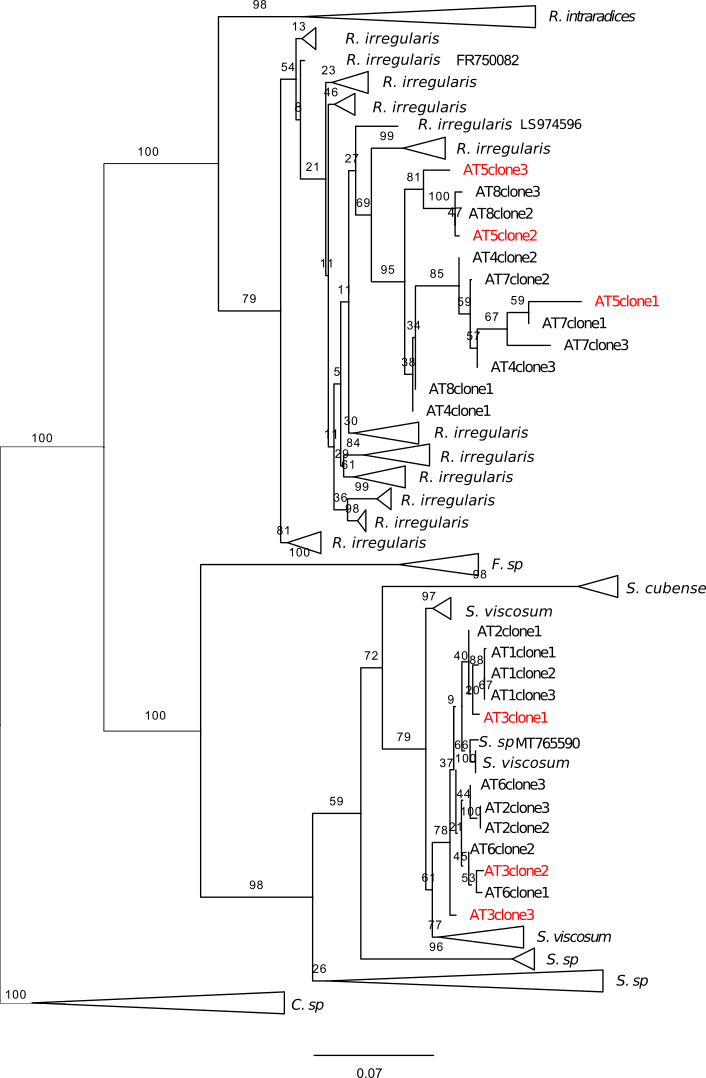


**Figure S4.** Phylogenetic maximum likelihood tree of the isolates AT from *A. tinctoria* and reference AMF species.  Characterised *Claroideoglomus* species were used as outgroup. Size of triangles represent the sequence numbers (vertically) and distances (horizontally). Two isolates used in this paper were highlighted with red colour.

**Mass-production of *Rhizophagus irregularis* and *Septoglomus viscosum***

The two AMF strains selected above (*Septoglomus* *viscosum* and *Rhizophagus irregularis*) were used to establish mass-production cultures with maize plants. Briefly, bleach soaked, and water washed maize seeds were germinated on sterilized lava substrate (120°C for 15 min) in a 5 L size pot. One-week old maize seedling was inoculated with AMF propagules of the two species (i.e., colonized *P. lanceolata* roots and substrates). Both AMF were used in association with *A. tinctoria* plants growing in the S-H cultivation system and in pots under greenhouse conditions (Experiment 2 and 3 in the main text).

***Alkanna tinctoria* acclimatization protocol**

*In vitro* produced *Alkanna tinctoria* plants (Cartabia et al., 2022) were acclimatized *ex vitro* as follows: plants were carefully removed from the culture medium (Figure S5a) and gently washed under running deionized water to remove the medium adhering to the roots. Subsequently, three to four rooted plants were transferred into glass pots (J. Weck GmbH u. Co. KG, Germany, 147x100 mm, size: 580 mL) closed by a cover and a cotton layer and containing a sterile (2x 121ºC for 15 min) substrate mixture [3 peatmoss (DCM, Grobbendonk, Belgium)/2 compost (DCM, Grobbendonk, Belgium)/1 perlite (Perligran Premium, KNAUF GMBH, Dortmund, Germany)/1 quartz 0.4-0.8 mm (no. 4, Euroquartz, Belgium)/1 quartz 1-2 mm (no. 1, Euroquartz, Belgium)] (Figure S5b). The plants were kept constantly moist in a growth chamber set at 24°C/20°C (day/night), RH of 80%, photoperiod of 16 h day^-1^ and PPF 130 μmol m^-2^s^-1^. (white fluorescent light, OSRAM L36 W830 Luminux). After 2 weeks, the glass pots were opened and placed inside a closed Sunbag (Sigma-Aldrich, Taufkirchen, Germany) (Figure S5c). After 1 week, the plants were transferred into single pots (7x7x6 cm) containing a sterile substrate mixture (2 peatmoss/2 compost/1 perlite /1 quartz 0.4-0.8 mm /1 quartz 1-2 mm) closed again under the Sunbag (Figure S5d). After 1 week, the Sunbags were gradually opened (2, 4, 6, 8 h) until the plants were completely adapted (±15 days) at the conditions in the growth chamber presented above (Figure S5e-g).

**
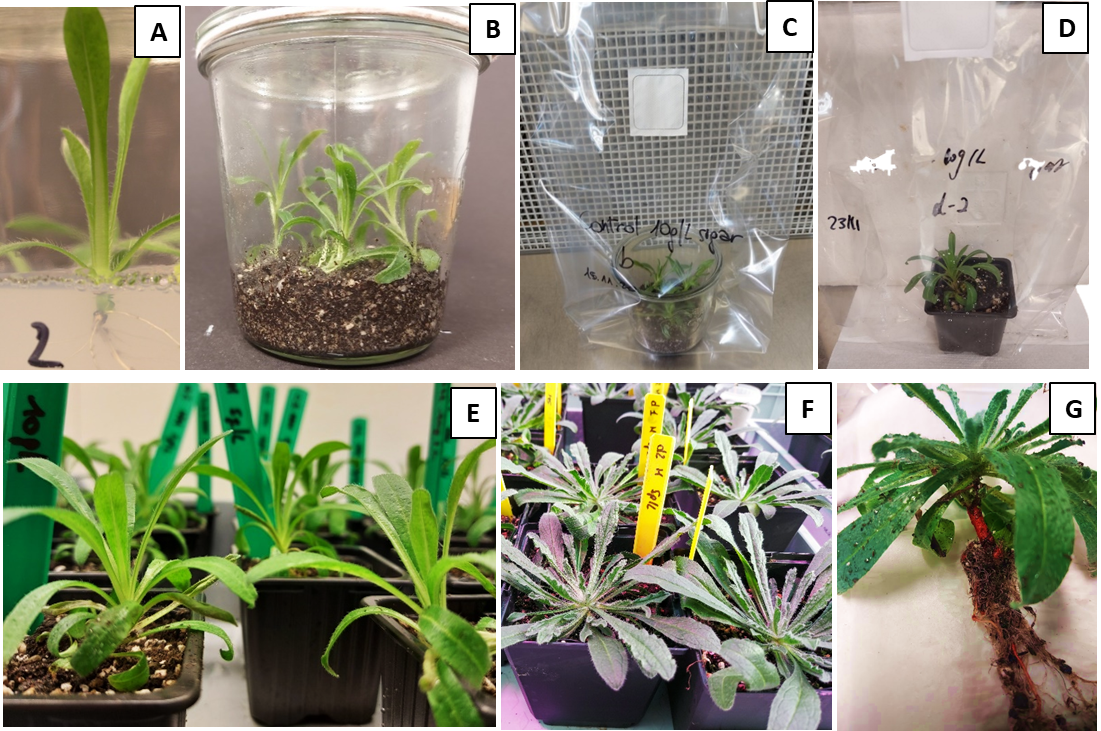
**

**Figure S5.** *Ex vitro* acclimatization and adaptation of *in vitro* produced plantlets of *A. tinctoria*: (**A**) rooted plantlets growing under *in vitro* conditions; (**B**) plantlets transferred in a closed glass pot filled with mixed sterile substrate; (**C**) glass pot opened inside a closed Sunbag; (**D**) plants transplanted into an individual pot in a closed Sunbag; (**E**) plants adapted to the growth chamber conditions; (**F**) plants transferred in bigger pots for the AMF-colonization and kept under greenhouse conditions (UCLouvain); (**G**) details of the characteristic roots’ reddish colour during the plants harvest.

**The modified Hoagland solution used in semi-hydroponic systems**

**Table S2**. Nutrients composition of the modified Hoagland solution, based on Hoagland and Arnon (1950), used in this study.

| **Chemical composition** | **Concentration [mg/L]** |
| --- | --- |
| **Macronutrients** |  |
| Ca(NO_3_)_2_·4H_2_O | 826 |
| KNO_3_ | 357 |
| KCl | 45.1 |
| K_2_SO_4_ | 105.4 |
| **Micronutrients** |  |
| KNO_3_ | 50 |
| KH_2_PO_4_ | 27.4 |
| MgSO_4_ | 120.4 |
| MnSO_4_·H_2_O | 0.5 |
| H_3_BO_3_ | 1.4 |
| CuSO_4_·5H_2_O | 0.2 |
| (NH_4_)_6_Mo_7_O_2­_·4H_2_O | 0.1 |
| ZnSO_4_·7H_2_O | 0.6 |
| **Iron** |  |
| Fe-EDTA | 19 |

**HPLC quantification: methodology validation**

**Response function**

The most adequate linear regression was selected with 95% expectation tolerance intervals included inside the ± 20% acceptance limits for each concentration level of the validation standards except the lowest one (Figure S6).

**Trueness, precision and accuracy**

Trueness was calculated at each concentration level of the validation standard and expressed in relative bias (RB). Relative bias was less than 3%, except for the lowest shikonin concentration.

Precision was evaluated intra-day (repeatability) and inter-day (intermediate precision) and expressed as relative standard deviations (RSD). The repeatability and the intermediate precision were less than 1.40% and 3.49%, respectively. All the trueness and precision results are in accordance with EMA guidelines criteria (≤ 15%).

Accuracy profiles, evaluating the sum of systematic and random errors of the test values (total error), are shown in Figure S7, indicating that the relative upper and lower 95% β-expectation tolerance limits are inside the acceptance limits, set at 20%, except for the lowest concentration. The method can thus be considered as accurate between 0.1 and 0.8 mg mL^-1^. The accuracy results are presented in Table S3.


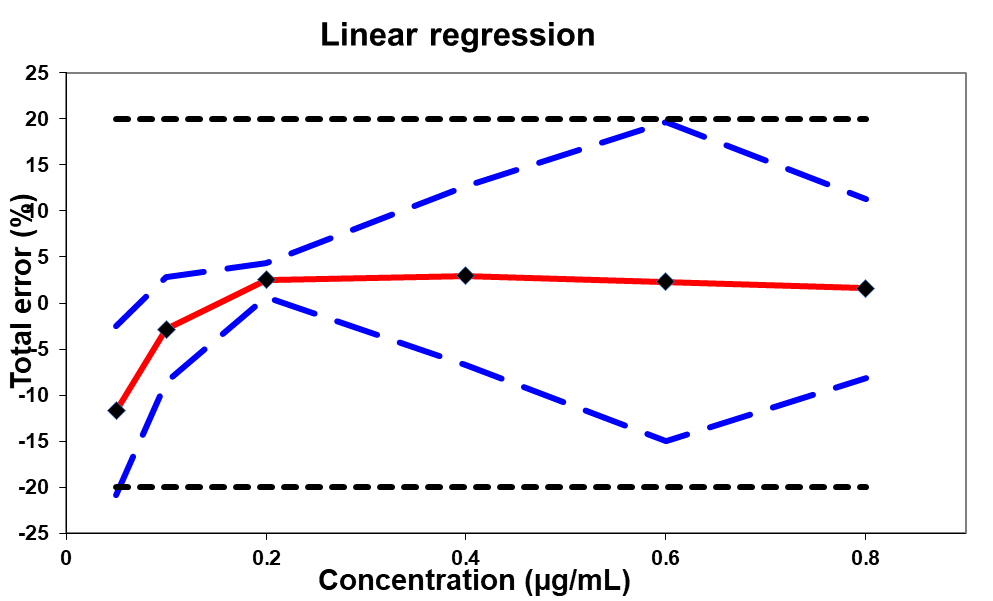


**Figure S6.** Accuracy profile of the mixture of shikonin obtained with linear regression. The plain line is the relative bias, dashed blue lines are the β-expectation tolerance limits (β = 95%) and dashed black lines represent the acceptance limits (± 20%).


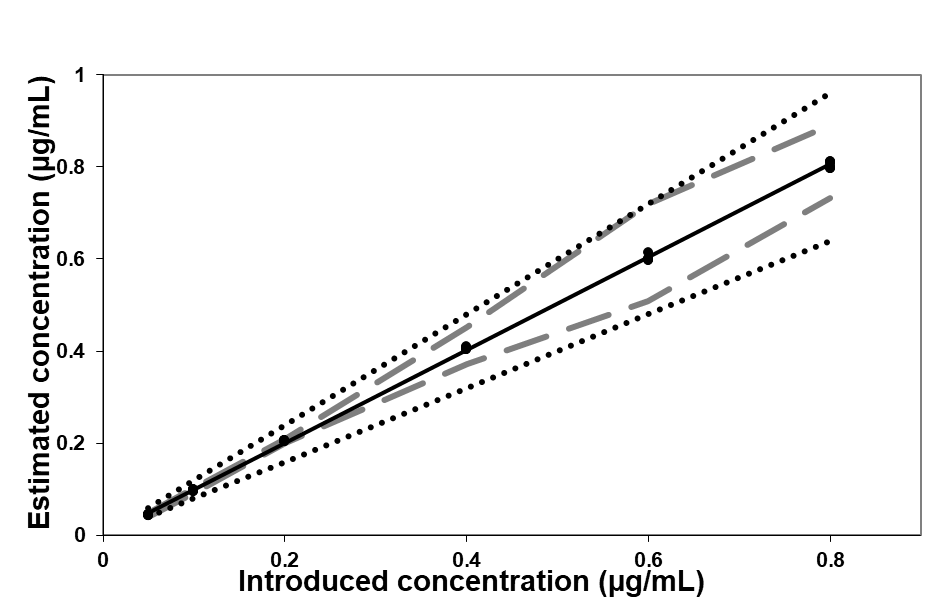


**Figure S7.** Linear profile of shikonin standard. The plain line is identity line (y = x), the dashed lines are the β-expectation tolerance limits (β = 95%) and dotted lines represent the acceptance limits (± 20%).

**Table S3.** Validation results obtained for the quantification of shikonin.

| **Validation criteria** | | **Concentration levels (µg mL^-1^)** | | | | | |
| --- | --- | --- | --- | --- | --- | --- | --- |
|  |  | 0.05 | 0.1 | 0.2 | 0.4 | 0.6 | 0.8 |
| **Response function** | | Linear regression | | | | | |
|  |  | Calibration range (5 points) | | | | | |
|  |  | 0.05-0.8 | | | | | |
| **Trueness** | Relative bias (%) | -11.68 | -2.84 | 2.53 | 2.98 | 2.31 | 1.58 |
| **Precision** | Repeatability (RSD %) | 0.34 | 0.72 | 0.31 | 1.40 | 0.76 | 0.53 |
|  | Intermediate precision (RSD %) | 1.85 | 1.58 | 0.51 | 2.71 | 3.49 | 1.97 |
| **Accuracy** (95 % relative β-expectation lower and upper tolerance limits in %) | | -20.85 | -8.55 | 0.71 | -6.76 | -14.97 | -8.15 |
|  |  | -2.52 | 2.85 | 4.36 | 12.71 | 19.6 | 11.31 |
| **Linearity** | Slope | 1.0252 | | | | | |
|  | Intercept | -0.0032 | | | | | |
|  | R^2^ | 0.9986 | | | | | |
| **Limit of detection** | | 0.0215 | | | | | |
| **Limit of quantification** | | 0.0651 | | | | | |

**Analysis of A/Sd target genes expression in *A. tinctoria* roots**

| **Table S4.** Primers used in the study. | | | |
| --- | --- | --- | --- |
| **Name (abbreviation)** | **Primer sequence 5’-3’ (forward)** | **Primer sequence 5’-3’ (reverse)** | **Accession number or reference used for primer designing** |
| *GAPDH* | ACCGTCCACTCCATTACCG | ATGAGGCAGCCCTTCCACC | Wu et al., 2008 |
| *GHQH/*G*10H* | ATTGCTGGGACTGATACAAC | CGTGCAACATCGGATTCTTC | MH077962 (Wang et al., 2019)  MN056184 (Song et al., 2020) |
| *LePGT1* | CTCTTAGGCTCCTCTGCT | CGTCGTCCACCTTATCTT | Wu et al., 2008 |
| *LePGT2* | AGAAAGGCAAGCAACCATC | CCCACCATCCAAATATTGCC | KT991522 (Liu et al., 2016)  AB055079.1 (Yazaki et al., 2002) |

**Alignments used to design primers**

Aligned GHQH sequences (MH077962 = *Arnebia euchroma*; MN056184 = *Lithospermum erythrorhizon*)*.* Coloured sequences are conserved regions that were used for primer designing.

10 20 30 40 50 60

----:----|----:----|----:----|----:----|----:----|----:----|

Consensus AUGGAAUACACAACAAUWUUGSUAGGSKUUUUSAUUGSUUWCGUUWUAUUUAAAGCUYUA

MH077962 AUGGAAUACACAACAAUAUUGGUAGGCUUUUUGAUUGGUUUCGUUUUAUUUAAAGCUUUA

MN056184 AUGGAAUACACAACAAUUUUGCUAGGGGUUUUCAUUGCUUACGUUAUAUUUAAAGCUCUA

70 80 90 100 110 120

----:----|----:----|----:----|----:----|----:----|----:----|

Consensus ACAAGAAAAUCAAAAAAYCUUCCUCCAGGUCCUCAUGUUCUUCCRAUUAUCGGWAACCUY

MH077962 ACAAGAAAAUCAAAAAACCUUCCUCCAGGUCCUCAUGUUCUUCCGAUUAUCGGAAACCUC

MN056184 ACAAGAAAAUCAAAAAAUCUUCCUCCAGGUCCUCAUGUUCUUCCAAUUAUCGGUAACCUU

130 140 150 160 170 180

----:----|----:----|----:----|----:----|----:----|----:----|

Consensus CACUUAGUUGGUAGCAUCCCUCAUAAAUCCAUCCUAAAACUUGCCGAAAAAUAUGGSCCA

MH077962 CACUUAGUUGGUAGCAUCCCUCAUAAAUCCAUCCUAAAACUUGCCGAAAAAUAUGGGCCA

MN056184 CACUUAGUUGGUAGCAUCCCUCAUAAAUCCAUCCUAAAACUUGCCGAAAAAUAUGGCCCA

190 200 210 220 230 240

----:----|----:----|----:----|----:----|----:----|----:----|

Consensus AUCAUGUCUUUACAAUUYGGKCARAUUCCAACWAUMGUYGUCUCAUCACCAAGCAUGGCG

MH077962 AUCAUGUCUUUACAAUUCGGUCAGAUUCCAACAAUCGUCGUCUCAUCACCAAGCAUGGCG

MN056184 AUCAUGUCUUUACAAUUUGGGCAAAUUCCAACUAUAGUUGUCUCAUCACCAAGCAUGGCG

250 260 270 280 290 300

----:----|----:----|----:----|----:----|----:----|----:----|

Consensus AAAGAAAUCCUCCAAAAACAGGAYRUUKCUUUCGCSGGGAAAAGAAUYCCCGACGCCCUC

MH077962 AAAGAAAUCCUCCAAAAACAGGAUGUUGCUUUCGCGGGGAAAAGAAUCCCCGACGCCCUC

MN056184 AAAGAAAUCCUCCAAAAACAGGACAUUUCUUUCGCCGGGAAAAGAAUUCCCGACGCCCUC

310 320 330 340 350 360

----:----|----:----|----:----|----:----|----:----|----:----|

Consensus AAUGCGCACAACCACUGGCAAUUCUCGGUWGUCUGGCUACCSGCGAAUUCCCUGUGGCGY

MH077962 AAUGCGCACAACCACUGGCAAUUCUCGGUAGUCUGGCUACCGGCGAAUUCCCUGUGGCGU

MN056184 AAUGCGCACAACCACUGGCAAUUCUCGGUUGUCUGGCUACCCGCGAAUUCCCUGUGGCGC

370 380 390 400 410 420

----:----|----:----|----:----|----:----|----:----|----:----|

Consensus ACRUUGAGRAAAAUAUUGACUUCCAAUAUWUUYACYAACAAUCGCCUCGAAGCUAGYCAR

MH077962 ACGUUGAGGAAAAUAUUGACUUCCAAUAUAUUCACCAACAAUCGCCUCGAAGCUAGUCAA

MN056184 ACAUUGAGAAAAAUAUUGACUUCCAAUAUUUUUACUAACAAUCGCCUCGAAGCUAGCCAG

430 440 450 460 470 480

----:----|----:----|----:----|----:----|----:----|----:----|

Consensus CACUUGAGGUCCCARAARGUYMGGGAUCUAGUAGAGUACUGCAAAAAAAGYGGGGAWAAA

MH077962 CACUUGAGGUCCCAGAAAGUCAGGGAUCUAGUAGAGUACUGCAAAAAAAGCGGGGAUAAA

MN056184 CACUUGAGGUCCCAAAAGGUUCGGGAUCUAGUAGAGUACUGCAAAAAAAGUGGGGAAAAA

490 500 510 520 530 540

----:----|----:----|----:----|----:----|----:----|----:----|

Consensus GGCGAGGCAGUCGAAAUMGGCCAGGCUGCYUACAGGACWUCCYUSAAYUUGUURUCUAGC

MH077962 GGCGAGGCAGUCGAAAUCGGCCAGGCUGCUUACAGGACUUCCCUCAACUUGUUGUCUAGC

MN056184 GGCGAGGCAGUCGAAAUAGGCCAGGCUGCCUACAGGACAUCCUUGAAUUUGUUAUCUAGC

550 560 570 580 590 600

----:----|----:----|----:----|----:----|----:----|----:----|

Consensus ACAAUMUUUUCCAAGGAYUUGGCGGAYUAYUAUWSUGAGACAGGUGCCCCAMGGGAGUUC

MH077962 ACAAUCUUUUCCAAGGAUUUGGCGGACUACUAUAGUGAGACAGGUGCCCCAAGGGAGUUC

MN056184 ACAAUAUUUUCCAAGGACUUGGCGGAUUAUUAUUCUGAGACAGGUGCCCCACGGGAGUUC

610 620 630 640 650 660

----:----|----:----|----:----|----:----|----:----|----:----|

Consensus AAAGAUGCAAUUUGGAACAUWUUGGUUGAGUCWGUKAAGCCAAAUUUGGCRGAYUUUKUU

MH077962 AAAGAUGCAAUUUGGAACAUUUUGGUUGAGUCAGUUAAGCCAAAUUUGGCAGAUUUUGUU

MN056184 AAAGAUGCAAUUUGGAACAUAUUGGUUGAGUCUGUGAAGCCAAAUUUGGCGGACUUUUUU

670 680 690 700 710 720

----:----|----:----|----:----|----:----|----:----|----:----|

Consensus CCAAUUCUUAGUAUGUUUGAYCUACARGGUAUUAAGCRACGUGCURGURUYCAUUUUGGA

MH077962 CCAAUUCUUAGUAUGUUUGACCUACAAGGUAUUAAGCGACGUGCUGGUAUCCAUUUUGGA

MN056184 CCAAUUCUUAGUAUGUUUGAUCUACAGGGUAUUAAGCAACGUGCUAGUGUUCAUUUUGGA

730 740 750 760 770 780

----:----|----:----|----:----|----:----|----:----|----:----|

Consensus AAGGGSCUCAAGAUUAUGGAARGUCUAGUYAAUGAACGUCUAGARCAYAGGGAARYMCAY

MH077962 AAGGGCCUCAAGAUUAUGGAAGGUCUAGUCAAUGAACGUCUAGAACACAGGGAAACCCAC

MN056184 AAGGGGCUCAAGAUUAUGGAAAGUCUAGUUAAUGAACGUCUAGAGCAUAGGGAAGUACAU

790 800 810 820 830 840

----:----|----:----|----:----|----:----|----:----|----:----|

Consensus GGUGCUACWCAUAAUGAUAUUUUGGAUAUMUUUCUCAAUUAUUGYGAUGAACAUCCSGAW

MH077962 GGUGCUACUCAUAAUGAUAUUUUGGAUAUCUUUCUCAAUUAUUGUGAUGAACAUCCGGAU

MN056184 GGUGCUACACAUAAUGAUAUUUUGGAUAUAUUUCUCAAUUAUUGCGAUGAACAUCCCGAA

850 860 870 880 890 900

----:----|----:----|----:----|----:----|----:----|----:----|

Consensus GAAMUUGAUCGUCACCGYGUYAAGCACACGAUCCUGGAUCUUUUU**AUUGCUGGGACUGAU**

MH077962 GAACUUGAUCGUCACCGCGUCAAGCACACGAUCCUGGAUCUUUUU**AUUGCUGGGACUGAU**

MN056184 GAAAUUGAUCGUCACCGUGUUAAGCACACGAUCCUGGAUCUUUUU**AUUGCUGGGACUGAU**

910 920 930 940 950 960

----:----|----:----|----:----|----:----|----:----|----:----|

Consensus **ACAAC**YUCUAGUGUGACYGAAUGGACMAUGGCAGAAUUAAUCMAGAAYCCMCAMGUSAUG

MH077962 **ACAAC**UUCUAGUGUGACUGAAUGGACCAUGGCAGAAUUAAUCCAGAAUCCACAAGUGAUG

MN056184 **ACAAC**CUCUAGUGUGACCGAAUGGACAAUGGCAGAAUUAAUCAAGAACCCCCACGUCAUG

970 980 990 1000 1010 1020

----:----|----:----|----:----|----:----|----:----|----:----|

Consensus AAAARGGCUAAAGAUGAGCUMKCACAAGUGAUYGGUAAAGGUAAAUSKUUA**GAAGAAUCC**

MH077962 AAAAGGGCUAAAGAUGAGCUCGCACAAGUGAUUGGUAAAGGUAAAUGUUUA**GAAGAAUCC**

MN056184 AAAAAGGCUAAAGAUGAGCUAUCACAAGUGAUCGGUAAAGGUAAAUCGUUA**GAAGAAUCC**

1030 1040 1050 1060 1070 1080

----:----|----:----|----:----|----:----|----:----|----:----|

Consensus **GAUGUUGCACG**UYUACCUUACCUACGUUGUAUAAUGAAAGAAGCCUUAAGGAAACAUCCW

MH077962 **GAUGUUGCACG**UUUACCUUACCUACGUUGUAUAAUGAAAGAAGCCUUAAGGAAACAUCCA

MN056184 **GAUGUUGCACG**UCUACCUUACCUACGUUGUAUAAUGAAAGAAGCCUUAAGGAAACAUCCU

1090 1100 1110 1120 1130 1140

----:----|----:----|----:----|----:----|----:----|----:----|

Consensus CCUGGUCCRUUUUUGUUUCCWCGAMGACCYGAGGAAGAYGUYGAAGUAGCSGGUUACACC

MH077962 CCUGGUCCGUUUUUGUUUCCACGACGACCUGAGGAAGAUGUCGAAGUAGCGGGUUACACC

MN056184 CCUGGUCCAUUUUUGUUUCCUCGAAGACCCGAGGAAGACGUUGAAGUAGCCGGUUACACC

1150 1160 1170 1180 1190 1200

----:----|----:----|----:----|----:----|----:----|----:----|

Consensus AUCCCGAAAGGGKCACAAGUACUYGUGAGCAUWUAUGCACUUGGYCGUGAYCCARASUCG

MH077962 AUCCCGAAAGGGGCACAAGUACUUGUGAGCAUUUAUGCACUUGGCCGUGAUCCAAACUCG

MN056184 AUCCCGAAAGGGUCACAAGUACUCGUGAGCAUAUAUGCACUUGGUCGUGACCCAGAGUCG

1210 1220 1230 1240 1250 1260

----:----|----:----|----:----|----:----|----:----|----:----|

Consensus UGGRAAGAUCCGUUARCAUUCAAKCCYGAGAGGUUUCUUGACUCGGARCUUGAYUUCCGA

MH077962 UGGGAAGAUCCGUUAGCAUUCAAGCCUGAGAGGUUUCUUGACUCGGAGCUUGACUUCCGA

MN056184 UGGAAAGAUCCGUUAACAUUCAAUCCCGAGAGGUUUCUUGACUCGGAACUUGAUUUCCGA

1270 1280 1290 1300 1310 1320

----:----|----:----|----:----|----:----|----:----|----:----|

Consensus GGCAACMAUUUCGARAUGUUGCCUUUUGGUGCYGGRAGAAGAUCAUGCCCUGGAUUGCCU

MH077962 GGCAACAAUUUCGAGAUGUUGCCUUUUGGUGCCGGAAGAAGAUCAUGCCCUGGAUUGCCU

MN056184 GGCAACCAUUUCGAAAUGUUGCCUUUUGGUGCUGGGAGAAGAUCAUGCCCUGGAUUGCCU

1330 1340 1350 1360 1370 1380

----:----|----:----|----:----|----:----|----:----|----:----|

Consensus AUGGCAGUUAGGAUGGUUCCUCUGMUUUUGGGGUCACUUAUCAACUCAUUUGAUUGGSWA

MH077962 AUGGCAGUUAGGAUGGUUCCUCUGCUUUUGGGGUCACUUAUCAACUCAUUUGAUUGGGUA

MN056184 AUGGCAGUUAGGAUGGUUCCUCUGAUUUUGGGGUCACUUAUCAACUCAUUUGAUUGGCAA

1390 1400 1410 1420 1430 1440

----:----|----:----|----:----|----:----|----:----|----:----|

Consensus CUKGAUGGUGGAAUGAAGCCUGAAGAUUURAGCAUGGAGGAAAAGGUKGGGCUYACAGCC

MH077962 CUUGAUGGUGGAAUGAAGCCUGAAGAUUUGAGCAUGGAGGAAAAGGUUGGGCUCACAGCC

MN056184 CUGGAUGGUGGAAUGAAGCCUGAAGAUUUAAGCAUGGAGGAAAAGGUGGGGCUUACAGCC

1450 1460 1470 1480 1490

----:----|----:----|----:----|----:----|----:----|-

Consensus CAAUUGGCUCAUYCWCURAAAAUUGUYCCAAYCCCWGUAAARGAAGAGUAA

MH077962 CAAUUGGCUCAUCCUCUGAAAAUUGUUCCAAUCCCAGUAAAGGAAGAGUAA

MN056184 CAAUUGGCUCAUUCACUAAAAAUUGUCCCAACCCCUGUAAAAGAAGAGUAA

Aligned LePGT2 sequences (KT991522 = *Arnebia euchroma*; AB055079.1 = *Lithospermum erythrorhizon*)*.* Coloured sequences are conserved regions that were used for primer designing.

10 20 30 40 50 60

----:----|----:----|----:----|----:----|----:----|----:----|

Consensus AKKWSYWSCAAACAAACMCAGCUMA**AGAAAGGCAAGCAACCAUC**AUGGAUUGAGAUKUAU

KT991522 AGUUCCAGCAAACAAACCCAGCUCA**AGAAAGGCAAGCAACCAUC**AUGGAUUGAGAUGUAU

AB055079.1 ATGAGTTCCAAACAAACACAGCTAA**AGAAAGGCAAGCAACCATC**ATGGATTGAGATTTAT

70 80 90 100 110 120

----:----|----:----|----:----|----:----|----:----|----:----|

Consensus UUGCCYMAAGARGUUCGRCCUUAUGCKCAYCUUGCAAGGYUAGACAAGCCUAUAGGCAGY

KT991522 UUGCCCCAAGAAGUUCGACCUUAUGCUCAUCUUGCAAGGUUAGACAAGCCUAUAGGCAGC

AB055079.1 TTGCCTAAAGAGGTTCGGCCTTATGCGCACCTTGCAAGGCTAGACAAGCCTATAGGCAGT

130 140 150 160 170 180

----:----|----:----|----:----|----:----|----:----|----:----|

Consensus UGGYURCUMGCUUGGCCMGCSUUYUGGUCCGUYGCAUUGRYUGCUGAUMUUGRMAGUCUA

KT991522 UGGUUGCUAGCUUGGCCAGCCUUCUGGUCCGUCGCAUUGGCUGCUGAUAUUGAAAGUCUA

AB055079.1 TGGCTACTCGCTTGGCCCGCGTTTTGGTCCGTTGCATTGATTGCTGATCTTGGCAGTCTA

190 200 210 220 230 240

----:----|----:----|----:----|----:----|----:----|----:----|

Consensus CCWAAAAUGKU**RGCAAUAUUUGGAUGGUGGG**CAGUUUGGAUCMGAGGUGCUGGAUGYACC

KT991522 CCUAAAAUGGU**AGCAAUAUUUGGAUGGUGGG**CAGUUUGGAUCAGAGGUGCUGGAUGCACC

AB055079.1 CCAAAAATGTT**GGCAATATTTGGATGGTGGG**CAGTTTGGATCCGAGGTGCTGGATGTACC

250 260 270 280 290 300

----:----|----:----|----:----|----:----|----:----|----:----|

Consensus AUUAAYGAUUACUUCGACCGCGAUUUYGAYAARAARGUGGAACGUACAAAAUCUAGACCW

KT991522 AUUAAUGAUUACUUCGACCGCGAUUUCGACAAGAAGGUGGAACGUACAAAAUCUAGACCU

AB055079.1 ATTAACGATTACTTCGACCGCGATTTTGATAAAAAAGTGGAACGTACAAAATCTAGACCA

310 320 330 340 350 360

----:----|----:----|----:----|----:----|----:----|----:----|

Consensus CUYGCUAGUGGCGCUGUCUCRCCWKCMMAAGGRYUSUGGUGGCUUGCWUUUCARCURUUY

KT991522 CUUGCUAGUGGCGCUGUCUCACCUUCCCAAGGAUUGUGGUGGCUUGCAUUUCAGCUGUUC

AB055079.1 CTCGCTAGTGGCGCTGTCTCGCCAGCAAAAGGGCTCTGGTGGCTTGCTTTTCAACTATTT

370 380 390 400 410 420

----:----|----:----|----:----|----:----|----:----|----:----|

Consensus AUUGGMUUGGGUGUUCUUUACCAAUUCAAYAUCUUGACUCUUGCAUURGCUAUYKUGCAU

KT991522 AUUGGCUUGGGUGUUCUUUACCAAUUCAACAUCUUGACUCUUGCAUUGGCUAUCUUGCAU

AB055079.1 ATTGGATTGGGTGTTCTTTACCAATTCAATATCTTGACTCTTGCATTAGCTATTGTGCAT

430 440 450 460 470 480

----:----|----:----|----:----|----:----|----:----|----:----|

Consensus GUKCCCYUUGUKUUUGCUUAYCCUCUCAUGAAAAGAAUUACMUAUUGGCCUCAAGCKUUU

KT991522 GUUCCCCUUGUUUUUGCUUAUCCUCUCAUGAAAAGAAUUACCUAUUGGCCUCAAGCUUUU

AB055079.1 GTGCCCTTTGTGTTTGCTTACCCTCTCATGAAAAGAATTACATATTGGCCTCAAGCGTTT

490 500 510 520 530 540

----:----|----:----|----:----|----:----|----:----|----:----|

Consensus CUUGGMGUWAUGAUMAGUUGGGGAGCUCUYUUAGGSUCCUCUGCYCUUAAAGGAAGUGUU

KT991522 CUUGGAGUAAUGAUCAGUUGGGGAGCUCUCUUAGGCUCCUCUGCUCUUAAAGGAAGUGUU

AB055079.1 CTTGGCGTTATGATAAGTTGGGGAGCTCTTTTAGGGTCCTCTGCCCTTAAAGGAAGTGTT

550 560 570 580 590 600

----:----|----:----|----:----|----:----|----:----|----:----|

Consensus GUYCCAAGYAKYGCCUACCCRCUYUACAUUUCGAGCUUUUUCUGGACUCUUGUUUAUGAU

KT991522 GUUCCAAGUAGCGCCUACCCGCUUUACAUUUCGAGCUUUUUCUGGACUCUUGUUUAUGAU

AB055079.1 GTCCCAAGCATTGCCTACCCACTCTACATTTCGAGCTTTTTCTGGACTCTTGTTTATGAT

610 620 630 640 650 660

----:----|----:----|----:----|----:----|----:----|----:----|

Consensus ACWAUYUAUGCACAUCAAGAUAAGGUMGACGAYGCAAAAGCWGGRAUUAAAUCCACWGCU

KT991522 ACUAUCUAUGCACAUCAAGAUAAGGUAGACGAUGCAAAAGCAGGGAUUAAAUCCACUGCU

AB055079.1 ACAATTTATGCACATCAAGATAAGGTCGACGACGCAAAAGCTGGAATTAAATCCACAGCT

670 680 690 700 710 720

----:----|----:----|----:----|----:----|----:----|----:----|

Consensus CUAMGMUUUGGWGAUGCWACMAARAUAUGGAUUASWUGGUUCGGMRUAGGAUGCAUUGSU

KT991522 CUAAGAUUUGGAGAUGCAACCAAGAUAUGGAUUAGUUGGUUCGGAGUAGGAUGCAUUGCU

AB055079.1 CTACGCTTTGGTGATGCTACAAAAATATGGATTACATGGTTCGGCATAGGATGCATTGGT

730 740 750 760 770 780

----:----|----:----|----:----|----:----|----:----|----:----|

Consensus GCUCUWSUUMUWGGSGGGYUCAUUGUSAACAUUGGGUUKCCUUAUUAYGURUUUKUGGCA

KT991522 GCUCUAGUUAUUGGGGGGCUCAUUGUGAACAUUGGGUUUCCUUAUUAUGUAUUUGUGGCA

AB055079.1 GCTCTTCTTCTAGGCGGGTTCATTGTCAACATTGGGTTGCCTTATTACGTGTTTTTGGCA

790 800 810 820 830 840

----:----|----:----|----:----|----:----|----:----|----:----|

Consensus AUYGCAACUGGUCAAUUGRYUUGGCAAAUUKUCACAGUUGAUUUAUCAUCUCCUAUGGAU

KT991522 AUCGCAACUGGUCAAUUGGCUUGGCAAAUUGUCACAGUUGAUUUAUCAUCUCCUAUGGAU

AB055079.1 ATTGCAACTGGTCAATTGATTTGGCAAATTTTCACAGTTGATTTATCATCTCCTATGGAT

850 860 870 880 890 900

----:----|----:----|----:----|----:----|----:----|----:----|

Consensus UGUGGYMRGAARUUYGUKUCYAACCAAUGGUUUGGUGCUAUWAUMUUCASYGGCAUCUUR

KT991522 UGUGGUCGGAAAUUCGUUUCUAACCAAUGGUUUGGUGCUAUUAUAUUCAGUGGCAUCUUG

AB055079.1 TGTGGCAAGAAGTTTGTGTCCAACCAATGGTTTGGTGCTATAATCTTCACCGGCATCTTA

910 920

----:----|----:----|--

Consensus SUUGGRAGAUUGUUUMCUUAGC

KT991522 CUUGGAAGAUUGUUUACUUAGC

AB055079.1 GTTGGGAGATTGTTTCCTTAG-

**References**

Cartabia, A., Sarropoulou, V., Grigoriadou, K., Maloupa, E., Declerck, S. (2022). *In vitro* propagation of *Alkanna tinctoria* Tausch.: a medicinal plant of the Boraginaceae family with high pharmaceutical value. *Industrial Crops and Products*. 182, 114860. https://doi.org/10.1016/j.indcrop.2022.114860

Cranenbrouck, S., Voets, L., Bivort, C., Renard, L., Strullu, D.-G., Declerck, S. (2005). “Methodologies for *in vitro* cultivation of arbuscular mycorrhizal fungi with root-organs” in *In vitro* culture of mycorrhizas, eds Springer-Verlag, Heidelberg, 341-375. doi: 10.1007/3-540-27331-X_18

Hoagland, D. R., Arnon, D. I. (1950). *The Water-Culture Method for Growing Plants without Soil* Vol. 347. Berkeley, CA: The College of Agriculture.

Katoh, K., Standley, DM. (2013). MAFFT multiple sequence alignment software version 7: improvements in performance and usability. *Mol Biol Evol.,* 30(4), 772–780. doi: org/10.1093/molbev/mst010

Krüger, M., Stockinger, H., Krüger, C., Schüßler, A. (2009). DNA-based species level detection of Glomeromycota: one PCR primer set for all arbuscular mycorrhizal fungi. *New Phytol*, 183(1), 212-223. doi: org/10.1111/j.1469-8137.2009.02835.x

Krüger, M., Krüger, C., Walker, C., Stockinger, H., Schüßler, A. (2012). Phylogenetic reference data for systematics and phylotaxonomy of arbuscular mycorrhizal fungi from phylum to species-level. *New Phytol*, 193(4), 970-984. doi: org/10.1111/j.1469-8137.2011.03962.x

Tan, L., Chao-Geng, Lv., Sheng, W., Wan-Zhen, Y., Lan-Ping, G. (2016). Transcriptome-based gene mining and bioinformatics analysis of p-hydroxybenzoate geranyltransferase genes in *Arnebia euchroma*. China journal of Chinese materia medica, 41(8), 1422-1429. doi: 10.4268/cjcmm20160809

Omar, M. B., Bolland, L., & Heather, W. A. (1978). A permanent mounting medium for fungi. *Stain technol.,* 53(5), 293-294. PMID: 87039

Schüßler, A., Walker, C. (2010). “The Glomeromycota. A species list with new families and new genera” in libraries at The Royal Botanic Garden Edinburgh, The Royal Botanic Garden Kew, Botanische Staatssammlung Munich, and Oregon State University. Electronic version freely available online at www.amf-phylogeny.com

Song, W., Zhuang, Y., & Liu, T. (2020). Potential role of two cytochrome P450s obtained from *Lithospermum erythrorhizon* in catalyzing the oxidation of geranylhydroquinone during Shikonin biosynthesis. *Phytochemistry*, 175, 112375. doi: org/10.1016/j.phytochem.2020.112375

Stamatakis, A. (2014). RAxML version 8: a tool for phylogenetic analysis and post-analysis of large phylogenies. *Bioinformatics (Oxford, England)*, 30(9), 1312–1313. doi:org/10.1093/bioinformatics/btu033

Wang, S., Wang, R., Liu, T., Lv, C., Liang, J., Kang, C., Zhou, L., Guo, J., Cui, G., Zhang, Y., Werck-Reichhart, D., Guo, L., Huang, L. (2019). CYP76B74 Catalyzes the 3′′-Hydroxylation of Geranylhydroquinone in Shikonin Biosynthesis. *Plant Physiol*, 179 (2), 402-414. doi: org/10.1104/pp.18.01056

Wu, S. J., Qi, J. L., Zhang, W. J., Liu, S. H., Xiao, F. H., Zhang, M. S., Xu, G. H., Zhao, W. G., Shi, M. W., Pang, Y. J., Shen, H. G., Yang, Y. H. (2009). Nitric oxide regulates shikonin formation in suspension-cultured *Onosma paniculatum* cells. *Plant Cell Physiol.*, 50(1), 118-128. doi: org/10.1093/pcp/pcn178

Yazaki, K., Kunihisa, M., Fujisaki, T., Sato, F. (2002). Geranyl Diphosphate:4-Hydroxybenzoate Geranyltransferase from *Lithospermum erythrorhizon*: cloning and characterization of a key enzyme in shikonin biosynthesis. *J. Biol. Chem*. 277(8), 2002, 6240-6246, doi : org/10.1074/jbc.M106387200
